# HIV prevention and HIV care among transgender and gender diverse youth: design and implementation of a multisite mixed-methods study protocol in the U.S.

**DOI:** 10.1186/s12889-019-7605-4

**Published:** 2019-11-15

**Authors:** Laura Jadwin-Cakmak, Sari L. Reisner, Jaclyn M. W. Hughto, Liz Salomon, Miguel Martinez, Elliot Popoff, Bré Anne Rivera, Gary W. Harper

**Affiliations:** 1 Department of Health Behavior & Health Education, University of Michigan School of Public Health, 1415 Washington Heights, Ann Arbor, MI 48109 USA; 2 Pediatrics, Boston Children’s Hospital and Harvard Medical School, 300 Longwood Ave, Boston, MA 02115 USA; 3 Department of Epidemiology, Harvard T.H. Chan School of Public Health, 677 Huntington Ave, Boston, MA 02115 USA; 4 The Fenway Institute, Fenway Health, 1340 Boylston Street, Boston, MA 02215 USA; 5 Departments of Epidemiology and Behavioral and Social Sciences, Brown University School of Public Health, 121 South Main Street, Providence, RI 02912 USA; 6 Center for Health Equity Research, Brown University School of Public Health, 121 South Main Street, Providence, RI 02912 USA; 7 Children’s Hospital Los Angeles, Center for Transyouth Health and Development, 4650 Sunset Blvd, MS#2, Los Angeles, CA 90027 USA; 8 Trans Sistas of Color Project, 77 Victor Street, Highland Park, MI 48203 USA

**Keywords:** Transgender, HIV prevention, HIV care continuum, Adolescent, Young adult, Mixed methods

## Abstract

**Background:**

In the U.S., transgender and gender diverse (TGD) populations face structural, interpersonal, and individual barriers to healthcare. Less is known, however, about the HIV prevention and treatment experiences of TGD youth in the U.S. The current study was developed to fill this research gap.

**Methods:**

This article describes the research protocol for a multi-site, U.S.-based mixed-methods study that sought to identify the multi-level facilitators and barriers that influence participation of TGD youth in various stages of the HIV prevention (e.g., pre-exposure prophylaxis uptake) and care continua. A sample of diverse TGD youth ages 16–24 was recruited from 14 U.S. sites. TGD youth participants completed a one-time, in-person visit that included an informed consent process, computer-based quantitative survey, and in-depth qualitative interview assessing experiences accessing HIV prevention and/or care services. Providers serving TGD youth were recruited from the same 14 sites and completed a one-time visit via phone that included informed consent, demographic questionnaire, and in-depth qualitative interview assessing their experiences providing HIV prevention or treatment services to TGD youth.

**Results:**

Overall, 186 TGD youth ages 16–24 and 59 providers serving TGD youth were recruited and enrolled from across the 14 U.S. sites. TGD youth participants had a mean age of 20.69; 77.3% youth of color; 59.7% trans-feminine; 15.5% trans-masculine; 24.9% non-binary; 53.6% family income under poverty level. Providers included medical and mental health providers as well as case manager/care coordinators, HIV test counselors, and health educators/outreach workers. Providers were 81.3% cisgender and 30.5% people of color. Successes with community-engagement strategies and gender-affirming research methods are reported.

**Conclusions:**

This study addresses critical gaps in current knowledge about the HIV prevention and care experiences of TGD youth. Findings have implications for the development of HIV interventions across levels to support the health and well-being of TGD youth. Future research is warranted to replicate and expand on lessons learned regarding recruitment and engagement of communities of TGD youth, including longitudinal designs to assess engagement across their developmental stages. Lessons learned working with TGD youth through developing and implementing the study protocol are shared.

**Trial registration:**

Registered on ClinicalTrials.gov on 05/20/2015 (NCT02449629).

## Background

Transgender and gender diverse adolescents and emerging adults – individuals whose gender identity differs from their sex assigned at birth or whose gender identity does not fall into conventional binary categories of male or female, hereafter referred to as “TGD youth” – have unmet health needs and experience many disparate negative health outcomes relative to cisgender (non-transgender) youth [1–5]. One health area of specific concern is the epidemiology of HIV infection. Young transgender women are one of the groups most highly impacted by the HIV epidemic in the U.S. [6–8]. The high prevalence of HIV infection is especially pronounced among transgender women of color [9]. Although data specific to adolescent transgender women of color are limited, recent self-reported rates of HIV among adult Black transgender women were 19%, and 4% for adult Latina transgender women [6].

Most HIV-related research among TGD populations has focused on transgender women, as they carry most of the HIV burden among TGD people. However, more recently there is increasing research on the sexual risk behaviors, STI history, and provider interactions of young transgender men indicating that this group is also at-risk of acquiring HIV, especially for transgender men who have sex with men [10–13]. While there is very little data on HIV risks or HIV prevalence among youth who are non-binary or gender diverse, gender nonconformity is associated with higher risk of victimization and abuse compared to gender conforming and binary-identified youth, and victimization and violence can increase one’s risk for HIV infection [14]. A recent review in the Journal of the International AIDS Society identified the need for research on factors affecting adherence to and retention in care among HIV-positive youth and adolescents from key populations, including transgender youth, as “urgent” [15]. HIV-focused research with TGD youth that includes a range of gender identities is needed to fill these gaps.

Limited research has specifically explored the experiences of TGD youth across the HIV prevention and care continua, including stages from prevention to diagnosis, and from care linkage to viral suppression. A study of young transgender women living with HIV who were linked to and engaged in medical care found that, compared to other behaviorally-infected youth, young transgender women reported higher prevalence of unemployment, lower educational attainment, and lower adherence to antiretroviral therapy (ART) [16]. Findings related to viral load were mixed. Compared to other behaviorally-infected cisgender youth, young transgender women were not more likely than behaviorally-infected cisgender youth to have a detectable viral load. However, among young transgender women, higher rates of unemployment and lower educational attainment were associated with a higher likelihood of having a detectable viral load. While this study offers initial insights into some barriers to HIV care engagement and positive health outcomes among young transgender women, many questions remain, including: what gender-specific barriers may exist to optimizing the HIV care continuum for TGD youth, in what ways broad social determinants of health impact TGD youth’s engagement in the HIV care continuum, and what factors allow some TGD youth to engage in the care continuum despite experiencing these gender-specific and broad barriers. Also unknown are the facilitators and barriers to engagement in HIV prevention services, including HIV testing and pre- and post-exposure prophylaxis (PrEP and PEP), among TGD youth. Mixed methods research that identifies the factors that shape TGD youth’s access to and receipt of HIV prevention and care services can be used to improve this population’s access to culturally appropriate and gender-affirming services across the HIV prevention and care continua.

The socioecological framework is useful to understanding the HIV continua experiences of TGD youth because it facilitates consideration of how individuals’ environments across multiple levels influence their experiences and health [17, 18]. Major systems of influence that coincide with the socioecological model include: sociocultural/policy (macrosystem) factors, institutional/community (exosystem) factors; interpersonal (microsystem) factors; and intrapersonal (individual/ontogenic) factors. Research suggests that TGD adults face numerous, multi-level barriers to healthcare and, ultimately, to achieving optimal health outcomes. These barriers include sociocultural/policy factors such as policies restricting access to transgender-inclusive health insurance coverage; institutional/community factors such as societal bias/stigma toward transgender individuals and those living with HIV; interpersonal factors such as discriminatory healthcare providers or employers; and intrapersonal factors such as the avoidance of healthcare due to internalized stigma, fear of discrimination, or cost [19]. Less is known about the multi-level facilitators and barriers to HIV prevention and care for TGD youth. Given the developmental level of the youth population of interest, the socioecological model allows for the holistic study of the HIV care continua experiences of TGD youth by assessing the multi-level contexts shaping their HIV-related prevention and care needs and identifying multiple-level intervention targets to improve TGD youth’s access to and uptake of services across the continua.

To understand the HIV continua among TGD youth, it is also useful to incorporate frameworks that are specific to TGD people’s health, such as Gender Minority Stress and Gender Affirmation. The Gender Minority Stress Model [20, 21], adapted from Meyer’s Minority Stress Model [22, 23], attributes health disparities associated with transgender and other gender minority identities to added stressors that come with membership in a stigmatized minority group, including “distal” stressors (e.g., lack of access to gender affirming care, discrimination in healthcare settings) and “proximal” stressors (e.g., anticipated stigma, internalized transphobia) [21]. The concept of gender affirmation refers to the process by which individuals are affirmed in their gender identity through social interactions [24]. The Gender Affirmation Framework posits that stigma leads to social oppression from a variety of sources (including in healthcare settings) and psychological distress, decreasing one’s access to gender affirmation while increasing one’s need for gender affirmation, which can lead to high risk contexts/risk behaviors. Constructs from these frameworks, including the need for and access to gender affirmation across different dimensions, experiences of social and economic oppression and marginalization, and psychological distress, can be used to better understand the lived experiences of TGD youth when accessing HIV prevention and care services.

This paper describes the development of the study protocol, research methods, and lessons learned from the U.S.-based “Affirming Voices for Action” study, a multi-site, mixed methods study that explored TGD youth's experiences of engagement in the HIV prevention and care continua. Grounded in the socioecological, gender affirmation, and gender minority stress models, the overarching study aims were to: 1) identify the multi-level facilitators and barriers that influence TGD youth’s engagement in the various stages of the HIV prevention and care continua; 2) create theoretical and empirical models to guide the development of interventions aimed at facilitating the full participation of TGD youth in the continua; and 3) develop recommendations and resources for healthcare and social service providers who work with TGD youth to improve the full inclusion of TGD youth in the continua of HIV prevention and treatment. Findings reported herein focus on describing the study protocol, characterizing community engagement and gender-affirming research methods, depicting the demographic and related characteristics of enrolled participants, and disseminating lessons learned working with TGD youth through developing and implementing the study protocol.

## Methods

This study was conducted through the Adolescent Trials Network for HIV/AIDS Interventions (ATN). In 2012, members of the ATN formed the Transgender Advisory Group (TAG) in order to address concerns related to the appropriate inclusion or exclusion of TGD youth within the ATN [25]. This working group included representatives in a wide variety of professional roles, including clinicians, study coordinators, outreach workers, and researchers. The TAG came together to share knowledge and provide guidance to the wider research network on how to create research and clinical environments that were inclusive of and affirming for TGD youth, as well as guidance on appropriate inclusion of TGD youth in research studies.

In 2014, TAG members proposed the current study, Affirming Voices for Action (AVA; officially titled ATN 130: Assessing the Engagement of Transgender and Other Gender Minority Youth Across the HIV Continuum of Care), in order to gather empirical evidence about the experiences of TGD youth across the HIV prevention and care continua that could be disseminated to research and clinical communities as well as used internally to improve care for and work with TGD youth within the ATN. To ensure that the diversity of TGD HIV-related healthcare experiences were documented, the research included youth with a range of identities, including trans feminine (i.e., individuals assigned male sex at birth who identify as women or another feminine identity) youth, trans masculine (i.e., individuals assigned female sex at birth who identify as men or another masculine identity) youth, and gender diverse or non-binary youth (i.e., individuals who identify as neither male or female, as both male and female, or as another gender identity that is not congruent with their assigned sex at birth), as well as youth from diverse racial and ethnic backgrounds, and from multiple geographic regions of the U.S.

### Study design

Engagement of TGD youth across the HIV prevention and care continua was assessed using a multiphasic mixed-methods study design. Both quantitative survey data and in-depth qualitative interview data were collected from TGD youth as well as from healthcare providers who had experience working with TGD youth. The mixed-methods design was utilized to gain greater insight into the research questions and to allow for data triangulation, lessening the limitations and biases inherent in one single research methodology [26, 27]. Qualitative and quantitative data on TGD youth were collected concurrently, reflecting a convergent parallel design, which permitted the collection of different but complementary data on the same topic in order to gain greater insight into a research problem than would be gained by using only one of the two methods in isolation [28]. The multiphasic study design allowed for an initial phase where secondary data analyses from a previous qualitative study with young transgender women (ATN 039 [29];) and a previous quantitative study on the HIV continuum among adolescents (ATN 086/106 [30];) were conducted and informed the development of the quantitative and qualitative measures for data collection. The qualitative portion of the study used a phenomenological investigative approach in order to help understand the sociocultural behaviors, language, roles, and interactions within a culture-sharing group (i.e., TGD youth). Phenomenology is specifically focused on describing what a given group of people have in common as they experience a particular phenomenon, and it is an inductive analytic approach that allows the patterns, themes, and categories of analysis to emerge from the data [27, 31].

### Community engagement research methods

The study was conducted in line with the community-based participatory research principles of cooperative and participatory engagement from stakeholders, collaborative participation, representation from community members, and dissemination of findings to stake-holders [32–34]. A Youth Advisory Board (YAB) was convened during initial protocol development with members at three geographically diverse sites: Detroit, Los Angeles, and Boston (3–4 youth per site). Advisory board members were ages 19–26 and were diverse in terms of racial, ethnic, and gender identities (e.g., genderqueer, trans, trans masculine, trans woman), and included youth living with HIV and youth not living with HIV. The advisory board was active throughout the duration of the study, providing insight and feedback on aspects of study design, measures and interview guide development, recruitment, and data interpretation. The advisory board participated in professional development and capacity-building trainings, and received payments for all study-related time and activities. The study also utilized the broader ATN youth advisory board to aid in the interpretation of the qualitative data and assure a culturally sensitive approach to the study of TGD youth. The employment of community feedback is especially beneficial when conducting research with understudied groups such as TGD youth as it ensures that the views of the culture-sharing group are integrated into the design, execution, and interpretation of the findings, as opposed to sole reliance on the researchers’ interpretations of the culture-sharing group’s experiences [26].

### Sample eligibility criteria

A sample of TGD youth and healthcare and social service providers serving TGD youth were recruited for the study from across the 14 U.S. sites: Tampa, FL; Los Angeles, CA; Washington, DC; Philadelphia, PA; Chicago, IL; New York, NY; New Orleans, LA; Miami, FL; Memphis, TN; Houston, TX; Detroit, MI; Baltimore, MD; Boston, MA; and Denver, CO.

Inclusion criteria for youth participants included: 1) not identifying solely with their sex assigned at birth (may identify as trans, transgender, trans woman, trans man, woman, man, gender non-binary, genderqueer, or any other gender, so long as their current gender identity and/or expression do not match their sex assigned at birth); 2) self-reports as between the ages of 16–24 years (inclusive) at time of consent; 3) able to understand both written and spoken English; 4) willing to participate in a computer-based survey and face-to-face interview about transgender and other gender minority identity and personal experiences seeking services across the HIV care continuum; and 5) willingness to provide signed informed consent for study participation. Given our focus on barriers and facilitators influencing participation with the HIV prevention and care continua, including primary HIV prevention and linkage to HIV care, the youth sample was stratified by HIV care status into two groups: TGD youth currently in HIV care (operationalized as self-report of positive HIV status and receipt of at least one HIVrelated service in the 6 months prior, whether at the study site or elsewhere), and TGD youth *not* currently in HIV care (operationalized as not having received any HIV-related services in the prior 6 months). An HIV-related service was defined as attending an HIV medical appointment inclusive of prescribing/monitoring ART, assessing viral load and CD4 count, or another service specific to HIV treatment. TGD youth not currently in HIV care could report any HIV status, including positive, negative, or unknown status.

Inclusion criteria for healthcare and social service providers interviewed included: 1) work experience as a medical provider, mental health professional, case manager, HIV test counselor, or health educator/outreach worker; 2) provides services at one of the 14 enrolling study sites/cities; and 3) works directly with or has formerly worked directly with TGD youth.

### Recruitment

Affirming Voices for Action was conducted through the ATN, which consisted of a network of researchers and 14 adolescent clinical sites located across the U.S. The study was centrally coordinated by the core research team located at the University of Michigan with assistance from the ATN Coordinating Center, and staff at each of the 14 study sites. The University of Michigan research team provided extensive training on qualitative interviewing to staff at each site, as well as training on cultural humility practices in working with TGD youth, use of the study screening tools, and implementation of the computer-based survey. Each site was in charge of local recruitment efforts, with the goal of enrolling 5–10 TGD youth currently in HIV care, 5–10 TGD youth not in HIV care, and 3–8 healthcare providers who worked with TGD youth. The involvement of all 14 sites in the study allowed for an adequate sample of TGD youth, a hard-to-reach population, without overburdening any particular site and allowing for a more geographically-diverse sample including regions of the country not often included in research on TGD populations.

Staff on the core research team included a Transgender Community Specialist, who identifies as a woman of trans experience and is a local and national advocate for transgender communities as well as people living with HIV. The Transgender Community Specialist assisted the sites with youth recruitment by identifying clinics and organizations that had programs or services specific to TGD youth in each city. These clinics and organizations were identified through her existing network of advocates across the U.S., conversations with members of advisory boards, additional colleagues from the TGD community, and extensive online research followed by phone and/or email follow up with identified organizations. In instances where study sites were not already connected with the identified transgender organizations in their area, the Transgender Community Specialist served as a liaison to provide information about the study and gain the organizations’ buy-in, then connecting them to staff at the local study site. TGD youth are a vulnerable population who often have interactions that are stigmatizing and not culturally sensitive; understandably, clinics and organizations serving TGD youth are sometimes hesitant to permit study recruitment of their clients. Having a member of our staff who was transgender-identified and who had extensive connections with transgender communities across the U.S. helped these clinics and organizations feel comfortable promoting the study to their TGD youth clients. The involvement of an advisory board of TGD-identified young people who assisted with the development of and approved final versions of the study survey and interview guides also helped local TGD-serving organizations feel comfortable working to promote the study.

In addition to facilitating new connections with TGD-serving organizations in each of the 14 cities, each site recruited potential youth participants through their patient population, existing community partners, existing youth community advisory boards, local HIV prevention coalitions, and outreach at local community events. Members of the study Youth Advisory Board and existing advisory boards at each site were asked to promote the study to TGD youth in their communities, and sites received Institutional Review Board (IRB) approval to provide a $10 recruitment incentive for each young person they referred. When permitted by their local IRB, sites also promoted the study via social media. The coordinating site created developmentally and culturally appropriate promotional materials including print and digital flyers and social media advertisements. Materials featured images of young people that were diverse in terms of gender expression and race/ethnicity with a youth-friendly design (e.g., vibrant colors and updated font style) and a study logo designed by YAB members, tailored with local contact information for each study site. The promotional materials were designed with advisory board input and feedback. These materials were disseminated to each site and local TGD-serving organizations. Promotional materials were also shared with the networks of the research team, the TAG, and national transgender organizations to increase awareness of the study.

To recruit healthcare providers who work with TGD youth, site staff provided information about the study to healthcare providers in their clinic who worked with TGD youth as well as other healthcare providers in their city who were known to provide services to TGD youth. The core research team and TAG provided site staff with additional suggestions of healthcare providers to reach out to in their city. The contact information of healthcare providers who met eligibility criteria and expressed an interest in participating in the study was provided to the Project Director.

### Study procedures

The study was approved by the IRB of each study site, as well as the University of Michigan. All but one site received permission from their IRB to waive parental consent from participants ages 16 or 17; the site that was not able to waive parental permission did not enroll participants under age 18. Youth participants completed all study activities at their local site or at an alternate community-based site with study staff. Youth who were approached by study staff or who contacted staff about the study were asked if they were interested in learning about the study; if interested, they were informed of the nature of the study, the information to be collected, and the assessments involved. Those who were interested in participating were asked to give verbal consent to undergo a brief screening to determine eligibility before enrolling into the study (written consent for the screening procedure was waived by all involved IRBs).

Written informed consent was obtained from all youth participants who screened eligible to participate. TGD youth completed the audio computer-assisted quantitative survey first, which took approximately 45 min, before participating in the in-depth qualitative interview, which lasted 1.5 h on average. All TGD youth participants completed both the quantitative and qualitative portions of the study. A short debriefing interview was conducted with each youth participant at the end of the visit to assess participant distress and, if necessary, connect them to any needed resources. TGD youth participants were provided transportation to and from the study site as well as incentives for their participation; the amount varied by study site according to their usual levels of compensation for research participation. Transportation costs for youth participants were also covered.

The Project Director or Study Coordinator at each site contacted interested healthcare provider participants by phone and screened them for eligibility. Written informed consent was waived for provider participants; verbal consent was obtained all providers who screened eligible to participate. Provider participants were interviewed by phone by one of two University of Michigan staff members; they then answered a brief demographic questionnaire. Qualitative interviews with healthcare providers lasted 45 min on average. Debriefing questions at the end of each interview assessed participants for potential distress related to the interview and, if desired, healthcare providers were provided resources related to providing services to TGD youth. Provider participants were compensated for their time with a $25 gift card.

The web-based quantitative survey for youth participants was hosted and stored on a secure University of Michigan server. The data were encrypted and included no identifiable information beyond a unique identifier to link the quantitative data to the qualitative data from the same participant. The data for completed surveys was downloaded by the Project Director each week and stored in a restricted folder in the University of Michigan School of Public Health file server (with firewall protection). Once downloaded, the data were expunged from the web server. Quantitative data were reviewed weekly to ensure that the survey software was working properly.

All interviews were recorded and transcribed. Audio recorded interviews were transmitted to the core research team via the secure server of Westat, the institution that handled study data management, where they were downloaded and stored in a restricted folder in the University of Michigan School of Public Health file server (with firewall protection). Throughout the collection of qualitative data, portions of each interview were listened to and checked for quality and consistency; additional supervision and feedback was provided to interviewers as needed. Once transcriptions were thoroughly reviewed for accuracy, the recordings were destroyed.

### Measures

The quantitative survey for TGD youth elicited information regarding the facilitators and barriers to engagement with each stage of the HIV care continua (i.e., prevention, diagnosis, linkage to care, engagement in care, retention in care, initiation of ART, and viral suppression). The quantitative survey also included several new measures developed for this study to assess gender-specific constructs related to TGD youth’s needs and experiences in healthcare settings. The quantitative survey was reviewed by Protocol Team experts in survey design with transgender and adolescent populations, and piloted with our Youth Advisory Board, to ensure the feasibility and acceptability of the instrument. When possible, we used measures that had been developed for and validated with adolescent populations. The constructs assessed on the quantitative youth survey are shown in Table 1; the quantitative survey in its entirety is available as supplemental material (see Additional file 1: ‘*ATN 130 Quantitative Survey’*).

**Table 1:** Quantitative Survey Measures

Area of Focus	Constructs Assessed
Demographics	Age, education, income, health insurance status, housing status, race/ethnicity, relationship status, access to healthcare services; intersex status [35]
Gender identity, sex assigned at birth, and gender expression	Two-step method to assess current gender identity and assigned sex at birth [36]; current nonconforming gender expression [37]; visibility of TGD status [35].
Sexual orientation and attraction	Sexual orientation identity [35, 38]; changes in sexual attractions [39].
Gender-related developmental milestones	First awareness of gender discordance, age of coming out, age of first hormone use [38].
Gender affirmation	Social gender affirmation (living full-time; pronoun), medical gender affirmation (types accessed; hormone access), legal gender affirmation (name change, gender marker change) [38].
HIV and STIs	Self-reported HIV status, HIV testing behaviors, STI screening, history of STIs.
HIV prevention^a^	Access to and utilization of primary HIV prevention services, including PrEP and PEP.
HIV-specific demographics^b^	Age at HIV diagnosis, mode of HIV acquisition, HIV serostatus disclosure, history/current engagement in HIV care, history/current medication status, medication adherence, access to and utilization of secondary HIV prevention services [40].
Mental health	Depressive symptoms (CES-D [41]); anxiety symptoms (GAD PHQ [42]); PTSD symptoms [43]; mental healthcare service utilization, family/friend support of gender status, intimate partner violence, child abuse prior to age 15, suicidal ideation, suicidal attempt, self-harm [35, 38, 44]; self-esteem [45].
Substance use/abuse	Substance abuse (CRAFFT [46]); frequency of use of various drugs in past 6 months; Injection drug use in past 6 months; use of prescription drugs not prescribed to you in past 6 months; lifetime receipt of drug or alcohol abuse treatment; current holder of medical marijuana prescription; substance use and sex [47].
Psychosocial risks and supports	History of incarceration, homelessness, family socioeconomic status/poverty, foster-care system engagement, sex work, substance abuse treatment history [35, 38, 44]; general social support [48].
Sexual behavior	Sexual activity (oral, anal, and vaginal sex) with cisgender male, transgender male, cisgender female, and transgender female partners. Items included number of partners, partners’ HIV serostatus, frequency of oral, anal, and vaginal sex with and without a barrier (condom or dental dam) (adapted from [47]); Reasons for sexual activity [49].
Discrimination and violence/victimization	Experiences of discrimination in day-to-day life in past year and month [50] adapted from [51]; experiences of anti-transgender violence and victimization (adapted from [52]).
TGD Stress Constructs	Internalized transphobia; transphobia in context of sexual encounters [38]; physical and emotional symptoms attributed to gender-related mistreatment [53]; general life stress; affective growth subscale [54].
Healthcare experiences	Responsiveness of healthcare services to gender-related needs [35]; need for and access to gender affirmation in healthcare settings, experiences of anticipated and enacted stigma in healthcare settings, experiences of stigma across the HIV continua of care (scales developed for this study with involvement of YAB).

^a^Asked only of TGD youth who reported not living with HIV or unknown HIV status

^b^Asked only of TGD youth who reported living with HIV

**Fig. 1:**
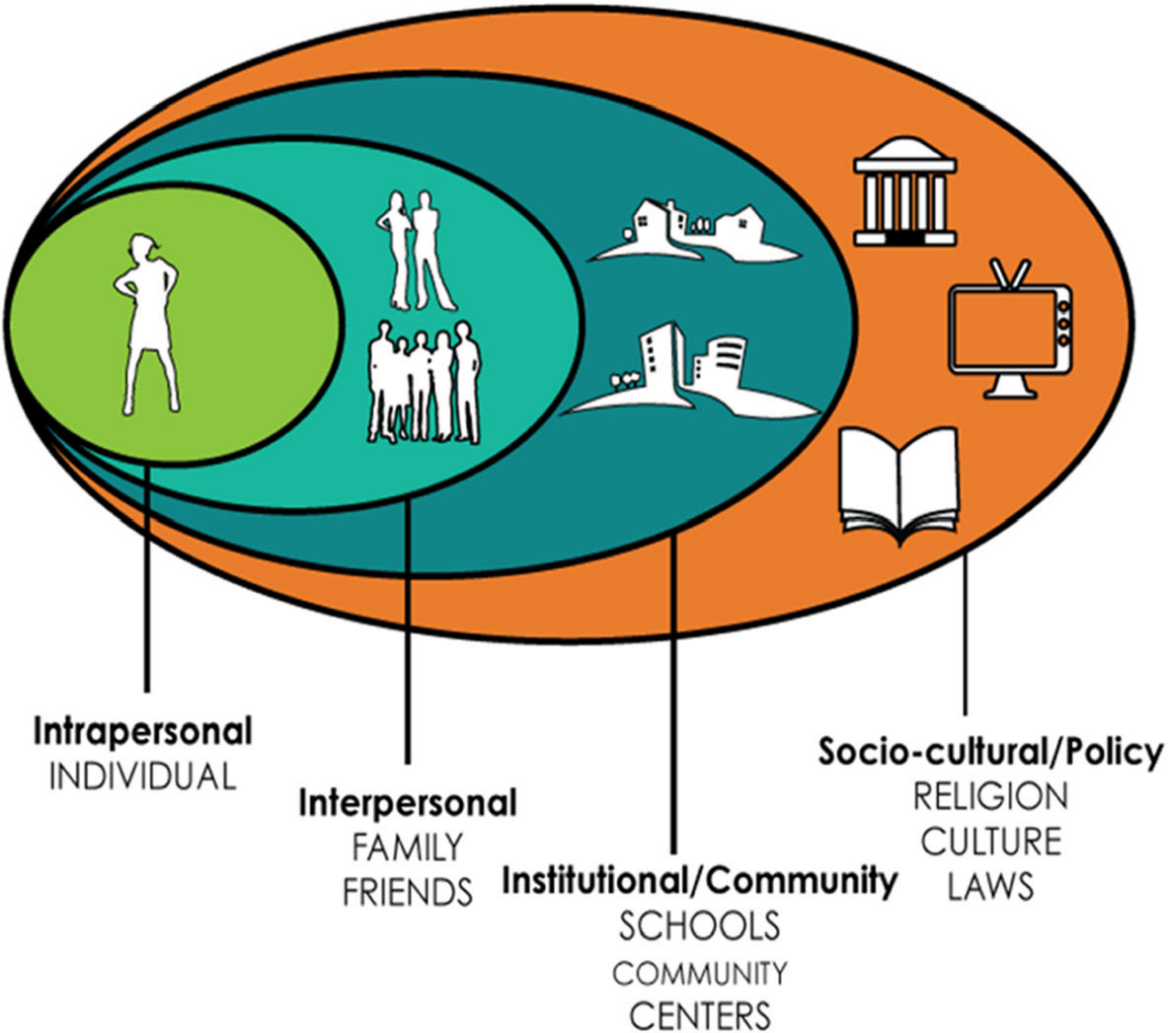
Socioecological Levels Visual Aid; Description: This figure, a graphic developed by our research team with feedback from the study Youth Advisory Board used as a visual aid during the in-depth interviews with TGD youth

The semi-structured qualitative interview guide for TGD youth was developed in collaboration with the Youth Advisory Board and Protocol Team using an iterative process of community feedback and revisions, as well as a secondary analysis of qualitative data from ATN 039 (see Table 2 for TGD Youth interview guide sections and example questions). Grounded in phenomenological and constructivist frameworks, the interview guide first asked participants to discuss their general life experiences as a transgender or gender diverse individual (using their own definitions, identity labels, and conceptualizations). Participants were prompted to explore their perceptions of the positive aspects of their gender identity that may contribute to resilience, followed by a discussion of their successes and challenges when seeking out and obtaining social, legal, and medical forms of gender affirmation. Participants were then guided through an in-depth exploration of their experiences engaging in the full continua of HIV prevention and care using a socio-ecological framework, (i.e., exploration of experiences at the intrapersonal, interpersonal, institutional/community, and socio-cultural/policy levels). A visual aid (see Fig. 1) was developed by the research team with feedback from the Youth Advisory Board to use in the qualitative interviews with TGD youth to describe the socioecological levels of relevance.

**Table 2:** IDI Guide for TGD Youth

Section	Example Questions
Introduction/Rapport Building	• How do you spend your time? • How do you identify in terms of gender? • At what age did you begin to identify as [gender identity]?
Resilience	• What are some of the things that help you deal with the stress that comes along with your gender identity? • What do you think are some of the positive things about being a person with your gender identity?
Gender Affirmation	[After providing introduction to social, medical, and legal gender affirming changes] • What changes, if any, have you made? • What have these changes been like for you?
General Healthcare Experiences	• Tell me about some experiences you’ve had while trying to use or while using healthcare services in general. • Tell me about any barriers you have experienced while using healthcare services, or while trying to use healthcare services. • Tell me about any facilitators or things that have helped you access healthcare services.
Primary HIV Prevention	• Tell me a little bit about your experiences with HIV prevention programs and services. • What things made it harder or kept you from using HIV prevention services? • What things helped you [would have helped you] use prevention HIV programs and services?
HIV Testing	• Tell me a little bit about your experiences with HIV testing. • What things made it harder or kept you from getting tested? • What things helped you or made it easier to get tested?
HIV Test Results	• Tell me a little bit about your experiences receiving HIV test results. • What made it more difficult to receive your results? • What things made it easier to receive your results?
HIV Continuum of Care^a^	Participants were asked about experiences, barriers, and facilitators across each of the following Continuum stages: Linkage to HIV Care, Engagement in HIV Care, Retention in HIV Care, Initiation of ART, Adherence to ART, and Viral Suppression.
Program Recommendations	• What kinds of programs, activities, or events would you like to see for transgender and gender diverse youth, or specifically for young people who share your gender identity? • What recommendations do you have for providers who work with transgender and gender diverse youth?

^a^Questions about the HIV Continuum of Care were asked only of TGD participants living with HIV

The semi-structured qualitative interview guide for providers was also developed in collaboration with the Youth Advisory Board and Protocol Team using an iterative process of community feedback and revisions. See Table 3 for Healthcare Provider Interview Guide sections and example questions. The guide, grounded in phenomenological and constructivist frameworks, began by eliciting information regarding participants’ experiences working with TGD youth. The interviewer then probed participants for their understanding of TGD youth’s barriers and facilitators to accessing each stage of the HIV prevention and care continua across four socioecological levels (i.e., intrapersonal, interpersonal, institutional/community, and socio-cultural/policy). The interview concluded by asking participants to provide recommendations on programs that are needed for TGD youth as well as recommendations for other providers on how to better serve TGD youth.

**Table 3:** IDI Guide for Healthcare Providers

Section	Example Questions
Introduction/rapport building	• Tell me about your experiences working with transgender and gender diverse youth.
Primary HIV Prevention^a^	• When it comes to accessing and using HIV prevention services and programs, what would you say is the typical experience of transgender and gender diverse youth? • How would you describe the quality of HIV prevention services provided to transgender and gender diverse youth? • What barriers make it difficult for transgender and gender nonconforming youth to make full use of HIV prevention services? • What facilitates transgender and gender nonconforming youth to fully access HIV prevention services?
HIV Testing & Diagnosis^a^	• When it comes to receiving the results of an HIV test, what would you say is the typical experience of transgender and gender nonconforming youth? • How would you describe the quality of the experiences transgender and gender nonconforming youth have receiving test results? • What barriers make it difficult for transgender and gender nonconforming youth to receive the results of their HIV test? • What facilitates transgender and gender nonconforming youth’s receipt of their HIV test results?
HIV Continuum of Care^a^	Participants were asked about experiences, quality of care, barriers, and facilitators of TGD youth across each of the following Continuum stages: Linkage to HIV Care, Engagement in HIV Care, Retention in HIV Care, Initiation of ART, Adherence to ART, and Viral Suppression.
Intervention/Program Recommendations	• What kinds of programs would you like to see for transgender and gender nonconforming youth? • What other resources (HIV-specific and not HIV-specific) would you like to see that would support you in your work with transgender and gender nonconforming youth? • What additional recommendations do you have for providers working with transgender and gender nonconforming youth?

^a^Healthcare Providers were asked only about those areas in which they reported personal experience working with TGD youth

### Quantitative analysis

Primary quantitative analyses will focus on descriptively characterizing the study sample of TGD youth in order to enhance qualitative data. This will include descriptively examining experiences of engagement in the HIV prevention and care continua in the sample by HIV serostatus. The Protocol Team will examine whether any subgroup differences exist by gender identity (TF vs. TM spectrum). Demographic, psychosocial, and other self-reported health-related indicators will be examined alongside the HIV prevention and care continua with an eye toward intervention development and delivery. Next, the Protocol Team will examine the specific barriers, facilitators, and correlates TGD youth face by HIV serostatus in relation to the HIV prevention and care continua. This will include elucidating factors unique to TGD youth in accordance with Gender Affirmation and Gender Minority Stress Frameworks—i.e., factors such as hormone use, gender-related developmental milestones (age first became aware of their trans identity, age of first hormone use), surgeries and body modification (pumping, risks with needle-sharing), transgender minority-related stigma and discrimination within healthcare and in multiple other domains. Differences will also be examined by age given developmental differences in healthcare and service engagement, and by race/ethnicity given racial/ethnic disparities in HIV and the HIV care continuum. For example, we will explore whether experiences of stigma in healthcare differ according to HIV status.

All quantitative analyses will be generated with a statistical significance at the alpha 0.05 level using SAS software, Version 9.4.1 of the SAS System for Microsoft Windows. Copyright© 2013 SAS Institute Inc. SAS and all other SAS Institute Inc. product or service names are registered trademarks or trademarks of SAS Institute Inc., Cary, NC, USA. Descriptive statistics (mean, standard deviation, frequencies, and proportions) will be estimated for all variables of interest. Distributions of individual items will be assessed, including missingness. The sample will also be stratified according to HIV status (positive vs. negative or unknown). Descriptive statistics on engagement in the HIV prevention continua will be examined for participants with an HIV negative or unknown status, while descriptive statistics for engagement in the HIV care continuum will be examined for HIV positive participants. Cross-tabs by gender identity category will be used to descriptively examine the distribution of variables across the HIV prevention and care continua. For outcomes that are independent of HIV status (e.g., stigma in healthcare, sexual risk behavior), cross tabs by HIV status (HIV positive vs. HIV negative or unknown status) will be explored. Bivariate and multivariable regression analyses will appropriately adjust for clustering due to recruitment across multiple geographic locations and study sites. Analyses will account for both natal sex (i.e., assigned sex at birth) and current gender identity.

### Qualitative analysis

Since this study seeks to learn about the range of facilitators and barriers that impact TGD youth’s engagement across the HIV care continuum, the primary qualitative analysis will utilize a psychological phenomenological framework [26, 27]. The research team will also employ aspects of deductive analysis that will involve the use of a priori codes, which represent critical constructs in the Socioecological model, Gender Affirmation Framework, and Gender Minority Stress Framework.

Data coding and analysis will be an iterative and interactive processes directed by the Protocol Chair and Project Director with participation from the broader research team. The qualitative data analysis team will hold regular meetings to review the developing set of themes. The team will first read all interview transcripts in order to increase familiarity with the data. Next, the team will assign a priori codes and create emergent codes. Through regular meetings, the qualitative data analysis team will develop a coding structure (i.e., a hierarchical set of constructs that account for the phenomena seen in the data). Once a codebook has been assembled, team members will apply to codes to transcripts via Dedoose software [55]. Team members will meet throughout the coding process to ensure the consistent application of codes.

Once the transcripts have been coded, team members will meet to identify consistent patterns in meaning, concepts, and themes across all interviews [31]. Data matrices will then be created as visual representations of the findings. Comparative analyses will be conducted to clarify differences that may exist for any subgroups of youth (e.g., by gender identity) in order to determine if and how recommendations and resources will need to be tailored to address sub-group specific topics. In addition, the research team will examine potential differences based on type of informant (i.e., youth vs. provider) to see if there are unique understandings of the lives of TGD youth. These comparative analyses will permit the qualitative data analysis team to specify resulting recommendations and resources that incorporate differences between groups as well as similarities among groups. Youth Advisory Board and community members who are not members of the Protocol Team will also be asked to review and provide feedback regarding the credibility of findings.

### Mixed-methods analysis

The study’s mixed-methods, convergent parallel design will allow for the triangulation of both qualitative and quantitative data, bringing together the strengths of these types of data by providing different but complementary perspectives on the same topic. The Protocol Team will use several approaches to integrate the qualitative and quantitative data. One method that will be used is data triangulation, whereby findings from one method will be validated or corroborated by using other methods, and the goal is to achieve a more accurate representation of a social phenomenon through this convergence [56]. This convergence may not always occur (such as in the case of conflicting or contradictory data), especially when working with understudied and oppressed/marginalized populations for whom quantitative methods may not be sufficient to capture a particular social phenomenon due to the lack of valid and reliable instruments. If confronted with a lack of data convergence, the research team will assess the underlying assumptions about the population and the types of data collected—a reflective process that will be beneficial in studying TGD youth given the multiple layers of oppression often experienced by these young people and the lack of empirical data documenting their lived experiences. Another method that will be used to address data divergence is to integrate multiple forms of data using data merging [57]. In cases where the qualitative and quantitative findings are divergent, the Protocol Team will also explore the possibility of using a complementary approach to data integration, whereby the goal is to attempt to connect and integrate parts, segments, or layers of a social phenomenon to validate or corroborate each other [58]. This is based on an assumption that the different methods do not provide the same representations of social realities, so there is not a desire to determine which method is a more accurate representation of reality than the other. Another approach will be to construct a multi-dimensional explanation of a phenomenon, based on the idea that social phenomena are multi-dimensional and thus may not be best represented by a single dimension alone [58]. This would allow the Protocol Team to ask different but intersecting questions with the qualitative and quantitative data, and allow for interdisciplinary perspectives on the social phenomena to be considered.

### Data analyses for the current article

For the purposes of this article, analyses focused on summarizing demographic and related characteristics of TGD youth and healthcare providers who participated in the AVA study protocol. Descriptive characteristics were tabulated in SAS 9.4.1.

## Results

### Study sample and characteristics

A total of 186 TGD youth were enrolled from the 14 U.S. participating ATN sites. Five youth did not complete key survey items (e.g., assigned sex at birth or current gender identity) and were therefore not included in the final quantitative data analytic sample. Demographic and related characteristics of TGD youth in the analytic sample (*n* = 181) are shown in Table 4. TGD youth had a mean age of 20.69 years (SD = 2.23 years) and were diverse in terms of sex assigned at birth (76.8% male, 23.2% female), gender identity spectrum (59.7% transgender women, 15.5% transgender men, 7.7% non-binary assigned female sex at birth, 17.1% non-binary assigned male sex at birth), race (77.3% youth of color), ethnicity (28.7% Latinx), sexual orientation (72.9% sexual minority), socioeconomic status (53.6% family income under poverty level, 45.3% lifetime participation in sex work, 50% lifetime experience of homelessness), and educational attainment (48.6% current students, 38.1% had high school degree or GED; 7.2% had college degree or more). Fifty-nine providers of TGD youth were enrolled and participated in an interview during this same 5-month period. Characteristics of healthcare and social services providers are presented in Table 5. Providers had a mean age of 41.8 years (SD = 11.2 years). 64.4% were cisgender female, 16.9% cisgender male, and 18.7% TGD. 30.5% were people of color. Providers were from various healthcare and social service professions, including medical providers (42.4%), mental health professionals (30.5%), case managers/care coordinators (18.6%), health educators/outreach workers (15.3%), and HIV test counselors (6.8%).

**Table 4:** TGD Youth Socio-demographics & related characteristics

	Total Sample	
	(*n* = 181)	
	100.00%	
	Mean	SD
Age in Years (range 16–24)	20.69	2.23
	*n*	%
Latinx/Hispanic Ethnicity	52	28.7
Race		
American Indian/Alaska Native	2	1.1
Asian/Native Hawaiian/Pacific Islander	4	2.2
Black or African American	93	51.4
White	41	22.7
Another Race	11	6.1
Multiracial	30	16.6
Sex Assigned at Birth		
Male	139	76.8
Female	42	23.2
Gender Identity		
Non-Binary, Assigned Female Sex at Birth (Genderqueer, Genderfluid)	14	7.7
Non-Binary, Assigned Male Sex at Birth (Genderqueer, Genderfluid)	31	17.1
Transgender female (Female, Transgender woman)	108	59.7
Transgender male (Male, Transgender Man)	28	15.5
Sexual Orientation		
Straight/Heterosexual	49	27.1
Gay/Lesbian/Same-Gender Attracted/Same-Gender Loving	62	34.3
Bisexual	13	7.2
Queer	11	6.1
Questioning/Not Sure	6	3.3
Asexual	2	1.1
Another Sexual Orientation	38	21.0
Low Family SES	97	53.6
Ward of the Court/State - Lifetime	35	19.3
Homeless - Lifetime	91	50.3
Current Student	88	48.6
Educational Attainment		
High School Degree or Less	61	33.7
High School Degree or GED	69	38.1
Some College or Technical School	38	21.0
College Degree or More	13	7.2
Sex Work - Lifetime	82	45.3
Location		
Northeast	27	14.9
Mid-Atlantic	30	16.6
Midwest	37	20.4
West	27	14.9
South	60	33.1

**Table 5:** Healthcare and Social Service Provider Demographics

	Total Sample	
	(*n* = 59)	
	100.00%	
	Mean	SD
Age in Years (range 23–63; *n* = 58)	41.8	11.2
	*n*	%
Latinx/Hispanic Ethnicity	8	15.7
Race		
American Indian/ Alaska Native	1	1.7
Asian/ Native Hawaiian/ Pacific Islander	2	3.4
Black or African American	12	20.3
White	41	69.5
Another Race	0	0
Multiracial	3	5.1
Gender Identity		
Cisgender Female	38	64.4
Cisgender Male	10	16.9
Non-Binary (Genderqueer, Genderfluid)	5	8.5
Trans-Feminine (Female, Transgender woman)	5	8.5
Trans-Masculine (Male, Transgender Man)	1	1.7
Professional Role^a^		
Medical provider	25	42.4
Mental health professional	18	30.5
Case manager/care coordinator	11	18.6
HIV test counselor	4	6.8
Health educators/outreach worker	9	15.3
Location		
Northeast	8	13.6
Mid-Atlantic	14	23.7
Midwest	6	10.2
West	10	16.9
South	21	35.6

^a^ Providers could indicate more than one professional role

### Lessons learned in gender-affirming recruitment and enrollment of TGD youth

Many factors contributed to the successful recruitment of TGD youth over a several month period. Simultaneous enrollment and implementation at 14 participating ATN sites was one factor that allowed the recruitment of this diverse sample of 186 TGD youth from July – December 2015. Sites each enrolled an average of 13.3 TGD youth participants (range = 6–21). The average total time each site spent enrolling participants was 2.9 months (range = 1.0–5.3 months), with sites enrolling an average of 5.4 TGD youth per month (range = 2.5–12.5).

Involvement of the transgender community from the very initial stages of the research was integral to the success of the study. Involvement included: TGD-identified study staff, including the Transgender Community Specialist who was a national advocate for transgender communities; a multi-site advisory board of TGD youth who provided feedback on all aspects of the study, including promotional materials and recruitment strategies; and guidance from the Youth Advisory Board and Protocol Team as well as study staff on survey and interview guide design. Recruitment was aided through the use of a multipronged recruitment approach that combined the study sites’ usual modes of recruitment with local outreach. Study sites’ usual modes of recruitment included recruiting from their patient population, existing community partners, and site-specific community advisory boards. Additionally, local outreach was conducted by the core study team to connect study sites with additional TGD-specific community organizations near each site; the study was promoted through social media channels; and study team, Youth Advisory Board, and Protocol Team networks were used to raise awareness of and enhance buy-in for the study. Sites varied in terms of their extant TGD patient population and established community connection(s) with TGD organizations; thus, these recruitment methods provided the needed assistance and resources for sites with less access to TGD youth populations.

Additionally, the present study took steps to ensure that participants would have an affirming experience participating in the study. Affirming participant experiences were achieved through the involvement of TGD youth advisers and community members, and TGD-identified staff in the development and refinement of the survey and interview guides. Additionally, study interviewers received training on cultural sensitivity practices with TGD youth and received ongoing feedback regarding language used and interactions with participants during practice interviews with mock participants and throughout the study (see description below). By creating an affirming research experience for TGD youth, participants felt more comfortable sharing information about the study with their networks after having participated, aiding in study recruitment efforts by spreading the word. The most impactful recruitment method was word of mouth from TGD youth who had participated in the research themselves and could attest to the gender-affirming research procedures and study staff.

### Best practices for training multiple sites and interviewer teams in TGD youth data collection and implementation

This study demonstrated the feasibility of training and overseeing mixed-methods data collection on sensitive TGD health topics from a large cohort of interviewers across 14 sites. Increasing the number of study sites is beneficial when working with hard-to-reach populations like TGD youth because it decreases the recruitment burden on any one individual site. However, it also poses potential challenges to collecting high-quality data, particularly qualitative data, which relies heavily on the individual skills of each interviewer. Interviewers for each site were selected from existing staff, and some site staff selected had little or no experience conducting qualitative interviews or working with TGD youth. To address these challenges, the study team developed an extensive training on qualitative research methods, qualitative in-depth interviewing, and working with TGD youth populations.

The initial training was delivered via two live webinar sessions, 2 h each, which allowed for interactive activities and answering trainees’ questions. The sessions were recorded for later reference and used to train several replacement interviewers due to personnel turnover at study sites. After the initial webinar training, interviewers received additional individual training and feedback on mock interview sessions conducted. Interviewers continued practicing and receiving detailed feedback from the Project Director and/or Project Coordinator until recorded mock interviews showed that the interviewer was consistently proficient in both the principles of qualitative interviewing and the use of affirming language with TGD youth. This required a minimum of 2 and a maximum of 5 mock interviews per interviewer, with training feedback calls with after each mock interview that lasted 30–60 min. Finally, as the interviews were conducted, portions of the audio recordings of interviews were reviewed for quality assurance, and additional feedback and training was provided to interviewers as needed throughout the study via telephone or video conference. This method of providing virtual training and supervision in both group and individual settings allowed for the cost-effective collection of high-quality qualitative data across multiple study sites and can be used to collect sensitive health information with other hard-to-reach and highly stigmatized populations.

## Discussion

### Study implications

This novel study addresses critical gaps in current knowledge of factors affecting U.S. TGD youth’s experiences across the HIV prevention and care continua. This research is the first, to our knowledge, to study the experiences of TGD youth within the HIV prevention and care continua and to identify barriers and facilitators to engaging TGD youth in HIV services in the U.S. Given the high prevalence of HIV and other sexually transmitted infections among TGD youth [6–8], and lack of access to inclusive, affirming quality healthcare reported by TGD [6, 59–61] the study findings could prove critical for improving HIV prevention and care services for TGD youth in the U.S., including increasing retention in care and viral load suppression for at-risk youth. This community-engaged research will ultimately assist providers to become better informed on how to better provide supportive and inclusive HIV services to TGD youth across the full range of HIV services. Findings from this study can also be used to inform the development of policy-, clinic-, and individual-level interventions to support the health and well-being of TGD youth including but not limited to HIV prevention and treatment.

Much of the research on transgender populations has focused on large metropolitan areas on the East and West Coasts of the U.S. [62–66], limiting our understanding of transgender communities from other geographic regions. Additionally, most studies with TGD populations that have focused on engagement in care have been comprised of adults over age 18, frequently with average age of the sample in the mid-30s or older, limiting our understanding of TGD youth populations’ experiences at this developmental stage [59, 67–70]. This study demonstrates the feasibility of collecting data from a relatively large sample of transgender and gender diverse youth in a short period from diverse geographic areas using a multisite design informed by community members at each decision-point of the protocol.

## Conclusion

In sum, this study protocol will provide vital information about the multi-level barriers and facilitators that TGD youth experience in needing, accessing, and receiving services across the HIV prevention and care continua. Study findings have implications for developing individual-, clinic-, and policy-level interventions to improve the health and well-being of TGD youth, and can inform best practices for providing HIV prevention services and HIV care to this population of traditionally underserved young people. Future research is warranted to replicate and expand on lessons learned to recruit and engage communities of TGD youth, including longitudinal designs to assess engagement in HIV prevention and care across adolescents’ and emerging adults’ development.

## Supplementary information

**Additional file 1.** Quantitative Survey; Description: This is the quantitative survey completed by TGD youth participants.

## Data Availability

The datasets generated during the current study are publicly available through the NICHD Data and Specimen Hub, https://dash.nichd.nih.gov/study/18119.

## Abbreviations

ART: Antiretroviral therapy
ATN: Adolescent Trials Network for HIV/AIDS Interventions
HIV: Human Immunodeficiency Virus
IRB: Institutional Review Board
PEP: Post-exposure prophylaxis
PrEP: Pre-exposure prophylaxis
TAG: Transgender Advisory Group
TGD: Transgender and gender diverse
