# Supplementary material for: HIV prevention and HIV care among transgender and gender diverse youth: design and implementation of a multisite mixed-methods study protocol in the U.S
Source: BMC Public Health. 2019 Nov 15;19:1531. doi: 10.1186/s12889-019-7605-4 (PMC6858737; doi:10.1186/s12889-019-7605-4)
Supplement: Supplementary file 1 — Additional file 1. Quantitative Survey; Description: This is the quantitative survey completed by TGD youth participants. [file 12889_2019_7605_MOESM1_ESM.docx]

**ATN 130 QUANTITATIVE SURVEY**

**1. INTRODUCTION**

Thank you for agreeing to participate in this very important study about adolescent health. To learn as much as possible about your health, you will be asked to answer questions about your background and your feelings and behaviors, including sexual and drug use behaviors. Some of these questions may be difficult for you to answer and dates may be hard to remember. Please take as much time as you need so that you give information that is as accurate as possible.

Some of these questions may seem embarrassing or very personal but remember, your answers are anonymous - that is, staff here will not see your answers, and your name will not be put on your answers. There are no right or wrong answers.

This interview is going to take around an hour to complete. If you have any questions at any point during the interview, just ask.

In some of the questions we will use the phrase “transgender and gender nonconforming.” When we say “transgender and gender nonconforming” we are referring to anyone whose current gender identity or gender expression is different from their assigned sex at birth.

**2. DEMOGRAPHICS**

**DemIntro** “The following questions are about your background, education, living situation and health behaviors. Some of the questions are very personal, but remember everything you answer is confidential, that is, your name will not be associated with any of the information you give. There are no right or wrong answers, so please respond as honestly as possible. You may also skip any question that you do not want to answer.”

**DemAge** “How old are you?”

**|_____|_____|** (0...99) Years ***(If 16-24, skip to DemGender)***

**DemAgeCheck** “You said that you were [%DemAge%], is that correct?”

1 Yes

2 No ***(Skip to DemAge)***

**DemGender** “What sex were you assigned at birth, meaning what the doctor put on your original birth certificate?”

1 Female

2 Male

**CurrGender** “What is your current gender identity?”

1 Female

2 Male

3 Trans female/Trans woman

4 Trans male/Trans man

5 Genderqueer/ Gender non-conforming

6 A gender not listed here (Please Specify: _____________)

**Pronoun** “What is your preferred pronoun?”

1 She/her

2 He/him

3 They/them

4 Ze/hir

5 Just my name please! (no pronouns)

6 Other (Please Specify: _____________)

**Intersex** “Have you ever been diagnosed with a medically-recognized intersex condition?”

1 Yes

2 No

3 Don’t Know/Not Sure

**DemHispanic** “Are you of Hispanic (Spanish) or Latino heritage?”

1 Yes

2 No ***(Skip to DemRace)***

**HispanicSpec** “Are you: (Choose only one. If you are of mixed Hispanic heritage, choose “Mixed Hispanic background.)”

1 Central American

2 Cuban

3 Dominican

4 Mexican, Mexican-American, or Chicano

5 Puerto Rican

6 Mixed Hispanic background

7 South American

8 Other (Please specify: ________________)

**DemRace** “In addition to knowing whether or not you are of Hispanic/Latino ethnic heritage, what is your racial background?” Please choose all that apply.

1 American Indian

2 Alaska Native

3 Asian

4 Native Hawaiian

5 Pacific Islander (other than Native Hawaiian)

6 Black or African American

7 White

8 Other (Please Specify: _____________)

**RelCurr “**Are you currently involved in a committed relationship with someone who you consider your boyfriend/girlfriend, spouse, or domestic partner? (Note: A committed relationship does not necessarily mean you are in a monogamous relationship.)”

1 Yes

2 No

**DemSchool** “Are you in school these days? “School” could mean a school or program where you are working toward a high school diploma, GED, or college/technical degree.”

1 Yes

2 No

3 No, I have graduated

4 Yes, but I am on summer/winter/spring break now

**DemGrade** “What is the highest level of education or grade you have completed?”

1 Eighth grade or less

2 More than eighth grade but did not complete High School

3 High School Graduate

4 GED

5 Some college/technical education

6 Technical School Graduate

7 College Graduate

8 Some graduate school

9 Master’s degree or above

10 None, no formal schooling

**DemIncDiverse** “Do your current sources of income or support include any of the following? Your sources of income may include public assistance, traditional, and non-traditional jobs. Check all that apply:”

1 Full-time employment/ Traditional job

2 Part-time employment/ Traditional job

3 Day labor

4 Income provided by family members

5 Income provided by a partner

6 Student stipend

7 Selling or dealing drugs

8 Sex work, survival sex or prostitution

9 Street income (panhandling, boosting, or stealing)

10 Unemployment benefits

11 SSI or disability

12 Food stamps

13 Income provided by a “sugar daddy” or “sugar mama”

14 Shelter, church, or other charitable organization

15 No income

16 None of these

17 Other (Please Specify: _____________)

**DemWhereLiveNow** “Where are you currently living or staying most of the time?”

1 Your own house or apartment

2 At your parents' house or apartment

3 At another family member’s house or apartment

4 At a non-family member's house or apartment

5 Foster home or group home

6 In a rooming, boarding, halfway house, or a shelter/welfare hotel

7 On the street(s) (vacant lot, abandoned building, park, etc.)

8 Some other place not mentioned (Please Specify: _____________)

**WhereHealthcare** “Where do you most often receive your health care services?”

1 County clinic

2 Community-based clinic (non-county department)

3 Private doctor's office

4 HMO (health maintenance organization)

5 School or college clinic

6 Emergency room

7 Mobile Health Team

8 Don't have regular source of healthcare

9 Don't seek healthcare **(*Skip to HealthInsur)***

10 Other (Please Specify: _____________)

**CareGender** “Please indicate the extent to which you agree or disagree with this statement:

The provider where I most often receive health care services is knowledgeable about transgender and gender nonconforming health.”

1 Strongly disagree

2 Somewhat disagree

3 Neutral

4 Somewhat agree

5 Strongly agree

**HealthInsur** “What kind of health insurance do you currently have? (Check all that apply)”

1 Medicaid or Medicare

2 Veteran's Administration

3 Private or work insurance (for example, Blue Cross)

4 School-based insurance

5 COBRA

6 No insurance

7 Other (Please Specify: _____________)

**NeededCareCost** “Was there a time in the past 6 months when you needed to see a doctor, but could not because of cost?”

1 Yes

2 No

**NeededCareGender** “In the past 6 months, have you had any problems getting health or medical services because of your gender identity or gender presentation?”

1 Yes

2 No

**3. GENDER AFFIRMATION, MILESTONES, AND GENDER PRESENTATION**

**GenderIntro** “The next set of questions are focused on gender identity, gender expression, and gender-related changes.”

**AgeRealize** “How old were you when you FIRST became aware that you were transgender or gender nonconforming, or felt your assigned sex at birth to be different than your gender identity?”

**|_____|_____|** Years-old

**AgeTransDisclose** “How old were you when you FIRST told another person about being transgender or gender nonconforming? (If this has not happened to you, please type in ‘this has not happened to me’)”

1 I was this age the first time I told someone that I was transgender or gender nonconforming. |_____|_____|

2 This has not happened to me. I have never told anyone I was transgender or gender nonconforming.

**SocialGenderAffirm** “Do you live full-time in your identified gender?”

1 Yes

2 No **(*Skip to MedicalGenderAffirm)***

3 This does not apply to me **(*Skip to MedicalGenderAffirm)***

**AgeSocialGenderAffirm** “How old were you when you FIRST began living full-time in your identified gender?”

1 **|_____|_____|** Years-old

2 I have never lived full time in my identified gender.

**MedicalGenderAffirm** “Have you accessed any medical interventions to affirm your gender (for example, hormones, surgeries to transition)?”

1 Yes

2 No, but I plan to ***(Skip to LegalGenderAffirm)***

3 No, and I do not plan to ***(Skip to LegalGenderAffirm)***

**AgeMedicalGenderAffirm** “How old were you when you FIRST sought out any form of medical gender affirmation services (i.e., hormones, surgery to transition)?”

**|_____|_____|** Years-old

**TypesMedicalAffirm** “Which medical interventions have you used to affirm your gender? (check all that apply)”

1 Pubertal blockers (to inhibit puberty)

2 Hormones (estrogen or testosterone)

3 Breast Implants (breast augmentation)

4 Chest Reconstruction/ Mastectomy

5 Breast Reduction (no mastectomy)

6 Facial or neck surgery (for example, nose job, cheek implants, forehead lift, trachael shave)

7 Abdominal surgery (hysterectomy, oophorectomy)

8 Lower feminizing surgery (vaginoplasty – creation of a vagina)

9 Lower masculinizing surgery (metoidioplasty, phalloplasty – creation of a microphallus or phallus)

10 Other (Please Specify: _____________)

***If did not select hormones in TypesMedicalAffirm, skip to SiliconeIntro.***

**NonRXhormoneIntro** “The next questions will ask you about injecting hormones that were not prescribed to you. Please answer honestly. Remember that your responses will not be viewed by clinic staff.”

**NonRXhormone1** “Have you ever injected hormones (or been injected by someone else with hormones) that were NOT given to you in a doctor's office or by a doctor or nurse?”

1 Yes 2 No ***(Skip to Silicone1)***

**NonRXhormone2** “When you injected hormones or were injected by someone else with hormones that were not given to you in a doctor's office or by a doctor or nurse, did you ever use a needle after someone else had already used it without correctly sterilizing it (for example, using bleach to clean it)? In other words, did you ever use a shared needle without cleaning it?”

1 Yes

2 No ***(Skip to Silicone1)***

**NonRXhormone3** “During the last 6 months, how many times have you injected hormones, or were injected by someone else, using a needle that someone else had used without cleaning it?”

1 0 times

2 1 or 2 times in the past 4 months

3 Once a month or less (3-4 times in the last 4 months)

4 2 or 3 times a month

5 1 or 2 times a week

6 3 to 5 times a week

7 Every day or almost every day

**SiliconeIntro** “The next questions will ask you about silicone injections ("pumping"). Please answer honestly. Remember that your responses will not be viewed by clinic staff.”

**Silicone1** “Have you ever injected silicone or been injected by someone else (for example at a “pumping party”)? We are not talking about an injection or shot given to you in a doctor’s office or by a doctor or nurse.”

1 Yes

2 No ***(Skip to LegalGenderAffirm)***

**Silicone2** “When you injected silicone or were injected by someone else, did you ever use a needle after someone else had already used it without correctly sterilizing it (for example by using bleach to clean it)? In other words, did you ever use a shared needle without cleaning it?”

1 Yes

2 No

**LegalGenderAffirm** “Have you legally affirmed your gender (i.e., name change, gender marker change on documents)?”

1 Yes

2 No

3 This does not apply to me

**Appear** “A person’s appearance, style, or dress may affect the way people think of them. On average, how do you think people would describe your appearance, style, or dress? (Choose one answer)”

1 Very feminine

2 Mostly feminine

3 Somewhat feminine

4 Equally feminine and masculine

5 Somewhat masculine

6 Mostly masculine

7 Very masculine

**Manner** “A person’s mannerisms (such as the way they walk or talk) may affect the way people think of them. On average, how do you think people would describe your mannerisms? (Choose one answer)”

1 Very feminine

2 Mostly feminine

3 Somewhat feminine

4 Equally feminine and masculine

5 Somewhat masculine

6 Mostly masculine

7 Very masculine

**VisibleNonconform** “People can tell I’m transgender or gender nonconforming even if I don’t tell them.”

1 Always

2 Most of the time

3 Sometimes

4 Occassionally

5 Never

**4. SEXUAL ORIENTATION AND ATTRACTION**

**SexOrIntro** “The next set of questions will focus on sexuality and sexual attraction.”

**SexOr** “What **best** describes your current sexual orientation?” (check one)

1 Straight/Heterosexual

2 Gay/Lesbian/Same-Gender Attracted/Same-Gender Loving

3 Bisexual

4 Queer

5 Questioning

6 I Do Not Label My Sexual Orientation

7 Unsure

8 Asexual

9 A Sexual Orientation Not Listed Here (Please Specify: _____________)

**LifetimeSex** “In your lifetime, with whom have you had sex (however you define it)?” (Choose all that apply.)

1 Cisgender/Non-Transgender Male (male at birth, identify as male)

2 Cisgender/Non-Transgender Female (female at birth, identify as female)

3 Transgender Man (FTM) (female at birth, identify as FTM, man, male)

4 Transgender Woman (MTF) (male at birth, identify as MTF, woman, female)

5 Female Assigned Sex at Birth Genderqueer/Gender Variant Person (female at birth, identify as genderqueer/gender variant)

6 Male Assigned Sex at Birth Genderqueer/Gender Variant Person (male at birth, identify as genderqueer/gender variant)

7 I have never had sexual contact

**ChangeAttractEver** “Have you ever experienced a change in attractions to others? (For example, feeling only attracted to women, then feeling attracted to both women and men.)”

1 Yes

2 No ***(Skip to AboutSex)***

**ChangAttractTrans** “Did you experience a change in attractions to others after recognizing you were transgender or gender nonconforming? (For example, feeling only attracted to women before transition, then feeling attracted to both women and men after transition.)”

1 Yes

2 No

**AboutSex** People have sex for many reasons. Below is a list of some reasons people have sex. What are some of the reasons you have had sex in your life? Please check all the reasons that have ever motivated your decision to have sex during your lifetime.

***(Pre-skip if LifetimeSex = 7)***

|  | Yes | No |
| --- | --- | --- |
| To make yourself feel better  To feel proud of yourself  Because you felt lonely  To feel prettier, sexier, or better looking  To brag to others  To provide something to yourself or others  To get back at someone you were angry with  Because you wanted to make it clear you loved or cared for someone  For the sake of curiosity  Because you were bored or needed a thrill  In exchange for money, drugs, or something else  Out of a sense that you owed your partners or that you had to have sex  To control your partner  Because you were afraid of losing your partner  To express closeness or intimacy  Just to relieve nerves  Just for your own pleasure  Just to please your partner |  |  |

**5. STI AND HIV TESTING AND DIAGNOSIS**

**STIandHIVIntro** “The next few questions ask about STIs and HIV. Remember these questions are confidential and will not be seen by clinic staff.”

**STIscreenever** “Have you EVER been screened by a doctor or other provider for a Sexually Transmitted Infection (STI), other than HIV? STIs include things such as Chlamydia, trichomoniasis (trich), syphilis, gonorrhea (clap), genital herpes, or genital warts (HPV).”

1 Yes

2 No ***(Skip to STIever)***

**STIscreennum** “How many times have you been screened for STIs during your lifetime?”

**|_____|_____|** Times

**STIscreenmonth** “When was your most recent STI screening? In what month and year? If you’re not sure, enter your best guess.”

**|_____|_____|** Month

**STIscreenyear**

**|_____|_____|** Year

**STIever** “Have you EVER been told by a doctor or other provider that you had a Sexually Transmitted Infection, other than HIV? STIs include things such as chlamydia, trichomoniasis (trich), syphilis, gonorrhea (clap), genital herpes, or genital warts (HPV), other than HIV?”

1 Yes

2 No ***(Skip toHIVtestever)***

**STIwhich** “With which sexually transmitted infection(s) were you diagnosed (check all that apply)?”

1 Anal warts (HPV)

2 Chlamydia

3 Genital warts (HPV)

4 Gonorrhea

5 Genital herpes

6 Syphilis

7 Other (Please Specify: _____________)

**HIVtestever** “Have you EVER been tested for HIV?”

1 Yes

2 No ***(Skip to PreventionEver)***

**HIVtestnum** “How many times have you been tested for HIV during your lifetime?”

**|_____|_____|** Times

**HIVrecentmonth** “When was your most recent HIV test? In what month and year? If you’re not sure, enter your best guess.”

**|_____|_____|** Month

**HIVrecentyear**

**|_____|_____|** Year

**HIVresult** “What was the result of your MOST RECENT HIV test?”

1 Positive

2 Negative

3 Unknown

4 Don’t Know

**NotReceived** “Have you ever been tested for HIV and NOT returned to receive your test result?”

1 Yes

2 No

***If HIVresult = 1, skip to DemAgePos.***

**6. PRIMARY HIV PREVENTION (not completed by youth living with HIV)**

**PreventionEver “**Have you ever accessed HIV prevention services or programs for example, risk reduction counseling, demonstrations on how to properly use condoms, programs for couples or groups focused on reducing the risk of acquiring HIV by changed behaviors)?”

1 Yes

2 No ***(Skip to PrepEver)***

**Prevention6Mos** “In the past 6 months, have you accessed HIV prevention services or programs (for example, risk reduction counseling, demonstrations on how to properly use condoms, programs for couples or groups focused on reducing the risk of acquiring HIV by changed behaviors)?”

1 Yes

2 No

**PreventionTrans** “When you have received HIV prevention services or programs, how specific were they to your needs as a transgender or gender nonconforming person?”

1 Very specific to transgender and gender nonconforming people

2 Mostly specific to transgender and gender nonconforming people

3 Somewhat specific to transgender and gender nonconforming people

4 Not at all specific to transgender and gender nonconforming people

**PrepEver** “Have you ever taken HIV medication before sex because you thought it would lower your chances of getting HIV (also known as PrEP)?”

1 Yes

2 No ***(Skip to PepEver)***

**Prep6Mos** “In the past six months, have you taken HIV medication before sex because you thought it would lower your chances of getting HIV (also known as PrEP)?”

1 Yes

2 No

**PepEver** “Have you ever taken HIV medication AFTER sex or potential exposure to HIV to lower your chances of getting HIV (also known as post-exposure prophylaxis or PEP)?”

1 Yes

2 No

***Skip to MentalHealthIntro.***

**7. HIV INFECTION, DISCLOSURE, & SECONDARY PREVENTION (only completed by youth living with HIV)**

**DemAgePos** “How old were you when you found out you were living with HIV?”

**|_____|_____|** (0...[%DemAge%]) Years

**DemRoute** “How do you think you got HIV?”

1 I was born with it

2 Use of blood products (blood, factor, etc.)

3 Sex with a male

4 Sex with a female

5 Sex with a transgender person

6 Injection drug use (for example, injecting into a vein, muscle, or under your skin (skin-popping)) not including drugs prescribed by your doctor to treat a medical condition

7 Sex with a male and injection drug use

8 Other (Please Specify: _____________)

9 Don't know

**ViralLoadKnown** “Do you know what your most recent viral load is?”

1 Yes

2 No ***(Skip to CD4Known)***

**ViralLoadSelfReport** “What is your most recent viral load?”

1 Undetectable (75 and under)

2 75 – 5,000

3 5,000-10,000

4 Above 10,000

**ViralLoadWhen** “How long ago did you have your blood drawn to learn your viral load?”

1 Less than 3 months ago

2 3-6 months ago

3 6-9 months ago

4 9-12 months ago

5 More than 12 months ago

**CD4Known** “Do you know what your most recent CD4 count is?”

1 Yes

2 No ***(Skip to HIVDisclose)***

**CD4SelfReport** “What is your most recent CD4 count?”

1 200 or under

2 200-1000

3 Greater than 1000

**CD4When** “How long ago did you have your blood drawn to learn your CD4 count?”

1 Less than 3 months ago

2 3-6 months ago

3 6-9 months ago

4 9-12 months ago

5 More than 12 months ago

**HIVDisclose** “Have you disclosed your HIV status to anyone?”

1 Yes

2 No ***(Skip to SecondaryPrev)***

**DiscloseWho** “Who have you told? (Choose all that apply)”

1 Current sex partner who is not a steady boyfriend or girlfriend

2 Past sex partner who is not a steady boyfriend or girlfriend

3 Current steady boyfriend (someone you knew for a while with whom you have an ongoing relationship)

4 Past steady boyfriend (someone you knew for a while with whom you had an ongoing relationship)

5 Current steady girlfriend (someone you knew for a while with whom you have an ongoing relationship)

6 Past steady girlfriend (someone you knew for a while with whom you had an ongoing relationship)

7 Friend

8 Mother

9 Father

10 Other relative

11 Priest/clergy

12 Other (Please Specify: _____________)

**SecondaryPrev** “In the past 6 months, have you accessed secondary prevention services or programs (for example, risk reduction counseling, demonstrations on how to properly use condoms, programs for couples or groups focused on reducing the risk of acquiring HIV by changed behaviors)?”

1 Yes

2 No

**8. MEDICATION ADHERENCE (only completed by youth living with HIV)**

**AdherenceIntro** “Now we’re going to ask you some questions about HIV medicines.”

**Adherence** “Have you ever received a prescription for pills or other medicines to treat your HIV?”

1 Yes

2 No ***(Skip to KeepAppt)***

**AdherenceMeds** “Are you currently taking pills or other medicines to treat your HIV?”

1 Yes

2 No ***(Skip to AdherenceNoTake)***

**AdherenceIntro2** “Many people with HIV have many pills or other medicines to take at different times during the day. Often people find it hard to always remember to take their pills or medicines. Sometimes people get busy and forget to carry their pills with them. Other times people find it hard to remember to take their pills like their doctor told them to, such as “with food” or “on an empty stomach” or “every 8 hours.” Other times people decide to skip pills to avoid side effects (like feeling sick to your stomach) or to just not take pills that day.

It is important for us to understand what people with HIV are really doing with their pills or medicines. Please tell us what you are actually doing. Don’t worry about telling us you don’t take all your pills or medicines. We want to know what is really happening, not what you think we want to hear.

The following questions ask you about the dose of pills that are prescribed for you. By “dose of pills,” we mean the quantity of pills or medicines prescribed to be taken at one particular time (for example, 3 pills before bedtime).

**AdherenceTimes** “How many times each day are you supposed to take a dose of medicine (pills or other medicines) to treat your HIV?”

1 Once a day

2 Twice a day

3 Three times a day

4 Four or more times a day

**AdherencePills** “What is the total number of pills your doctor has told you to take each day?”

**|_____|_____|** Pills

**AdherenceWeekend** Now we’re going to ask you to think about last weekend (Friday, Saturday and Sunday). Sometimes taking medication can be even more difficult on weekends. Thinking about the last weekend, how many doses did you miss? (Please type in a number from 0 (which means you didn’t miss any doses last weekend) to as many doses as you might have missed).

**|_____|_____|** Doses

***Note for programming:*** For **AdherenceWeekend,** allow responses from 0 to 12. If response is greater than 12, please display, “You have entered an invalid response. Please re-enter.”

For answers greater than 9, display, **AdherenceWeekendCheck** “I want to confirm the number of doses of pills that you missed taking last weekend. The number that you entered is displayed on the screen. Is that correct?”

1 Yes

2 No (Skip to AdherenceWeekend)

**AdherenceWeek** “Thinking about the last 7 days, about how many times did you miss taking a dose of pills? Please type in a number from 0 (which means you didn’t miss any doses of pills) to as many times as you might have missed taking a dose of pills.”

**|_____|_____|**  Times

***Ask AdherenceNoTake and AdherencePrescribed only if response to AdherenceMeds = 2. Else skip to KeepAppt.***

**AdherenceNoTake** “Has your doctor offered or prescribed pills or other medicines for your HIV infection but you never started taking those medicines?”

1 Yes

2 No

**AdherencePrescribed** “Have you been prescribed pills or other medicines for your HIV infection that you took for a while but have now stopped completely?”

1 Yes

2 No

**KeepAppt** “In the past 6 months, how many doctor’s appointments have you attended?”

**|_____|_____|** (0..80) Appointments

**MissedAppt** “In the past 6 months, how many doctor’s appointments have you missed?”

**|_____|_____|** (0..80) Appointments

**9. MENTAL HEALTH ISSUES AND TREATMENT**

**MentalHealthIntro** “The next questions ask you about your mental health and social history. Some of the questions are very personal, but remember everything you answer is confidential, that is, your name will not be connected to any of the information you give. Please respond as honestly as possible. You may also skip any question that you do not want to answer.”

**HHUIntro** “The next section asks about your use of mental health services in the past 12 months. Think of all the services you have received related to your mental health regardless of where or at which agency you received them.”

**HHUHelp** “In the past 12 months, did you want or need help with personal or family problems from a mental health professional such as a social worker, psychiatrist, psychologist or counselor?”

1 Yes

2 No

**HHUPsych** “Now I would like to ask you if, in the past 12 months, you have seen a psychiatrist, psychologist, marriage & family therapist, or social worker about the way you were feeling or behaving?”

1 Yes

2 No ***(Skip to HHUMeds)***

**HHUCounsel** “In the past 12 months, how many times have you gone for counseling?”

**|_____|_____|** (0..50) Times

**HHUMeds** “In the past 12 months, how many times has a health care professional prescribed any medication to help you sleep or cope with emotional or mental health problems you were having?”

**|_____|_____|** (0..50) Times

**IPV** “Have you ever been slapped, punched, kicked, beaten up, or otherwise physically or sexually hurt by a boyfriend/girlfriend, spouse, or some other intimate partner?”

1 Yes

2 No

3 Don't know/not sure

4 Prefer not to answer

**Abuse** “Were you ever physically or sexually abused as a child under age 15 years-old?”

1 Yes, physically abused

2 Yes, sexually abused

3 Yes, both

4 No

5 Don't know/not sure

6 Prefer not to answer

**SuicideConsiderEver** “In your lifetime did you ever seriously consider attempting suicide, that is, taking some action to end your own life?”

1 Yes

2 No ***(Skip to SelfHarmEver)***

**SuicideAttemptEver “**In your lifetime, have you ever attempted suicide, that is, try to kill yourself?”

1 Yes

2 No *(****Skip to SelfHarmEver)***

**SuicideResultEver** “Did a suicide attempt ever in your life result in an injury, poisoning or overdose that had to be treated by a doctor or nurse?”

1 Yes

2 No

**SelfHarmEver** “Have you ever in your lifetime intentionally harmed yourself (i.e., cutting, burning, hitting) without lethal intent, that is, without wanting to kill yourself?

1 Yes

2 No

**10. INCARCERATION, LIVING SITUATION, AND SEX WORK**

**JailIntro** “Some young people have had experiences with being arrested or put in jail. I would like to ask you a question about time that you may have spent incarcerated (in jail) or in juvenile detention (juvy).”

**JailEver** “Have you ever been incarcerated (been put in jail or prison, or held overnight after an arrest) or put in juvenile detention (juvy)?”

1 Yes

2 No

**HomelessEver** “Have you ever been homeless in your lifetime?”

1 Yes

2 No ***(Skip to Poverty)***

**HomelessRecent** “In the last 6 months, were you homeless at any time?”

1 Yes

2 No

**Poverty** “Have you or your family ever received assistance from the Aid to Families with Dependent Children (AFDC), Temporary Assistance for Needy Families (TANF), food stamp, or free lunch programs?”

1 Yes

2 No

3 Don't know

**Foster** “Have you ever been a ward of the court/state (DCFS, foster system, court-appointed group home, etc.)?”

1 Yes

2 No

3 Don't know

**SexWorkEver** “People define sex work in many different ways. For the following questions when we say sex work, we mean trading sexual activity or favors for food, money, a place to sleep, drugs or other goods.

Have you ever traded sexual activity or favors for food, money, a place to sleep, drugs or other material goods?”

1 Yes

2 No ***(Skip to CESDIntro)***

**SexWorkReasonEver** “What was your primary reason for trading sex? Choose one answer.”

1 To earn money to survive

2 To earn money in general

3 To earn money for gender enhancement surgery and/or hormones

4 Could not get another job

5 To trade for food, shelter, or drugs

6 Had no other options for getting money

7 It was a good job

8 I enjoy doing sex work

9 Other (Please Specify: _____________)

**SexWorkCurrent** “During the past 6 months, have you traded sexual activity or favors for food, money, a place to sleep, drugs or other material goods?”

1 Yes

2 No ***(Skip to CESDIntro)***

**SexWorkReason6mo** “In the last 6 months, what was your primary reason for trading sex? Choose one answer.”

1 To earn money to survive

2 To earn money in general

3 To earn money for gender enhancement surgery and/or hormones

4 Could not get another job

5 To trade for food, shelter, or drugs

6 Had no other options for getting money

7 It was a good job

8 I enjoy doing sex work

9 Other (Please Specify: _____________)

**11. MENTAL HEALTH SYMPTOMS & WELL-BEING**

**CESDIntro** “Please indicate how often you have felt this way during the past week including today.”

|  | During the past week: | Rarely or none of the time (less than 1 day) | Some or little of the time (1-2 days) | Occasionally or a moderate amount of time (3-4 days) | Most or all of the time (5-7 days) |
| --- | --- | --- | --- | --- | --- |
| **CESDa1** | I was bothered by things that usually don’t bother me. | 0 | 1 | 2 | 3 |
| **CESDa2** | I did not feel like eating;  my appetite was poor. | 0 | 1 | 2 | 3 |
| **CESDa3** | I felt that I could not shake off the blues even with help from my family. | 0 | 1 | 2 | 3 |
| **CESDa4** | I felt that I was just as good as other people. | 0 | 1 | 2 | 3 |
| **CESDa5** | I had trouble keeping my mind on what I was doing. | 0 | 1 | 2 | 3 |
| **CESDa6** | I felt depressed. | 0 | 1 | 2 | 3 |
| **CESDa7** | I felt that everything I did was an effort. | 0 | 1 | 2 | 3 |
| **CESDa8** | I felt hopeful about the future. | 0 | 1 | 2 | 3 |
| **CESDa9** | I thought my life had been a failure. | 0 | 1 | 2 | 3 |
| **CESDa10** | I felt fearful. | 0 | 1 | 2 | 3 |
| **CESDb1** | My sleep was restless. | 0 | 1 | 2 | 3 |
| **CESDb2** | I was happy. | 0 | 1 | 2 | 3 |
| **CESDb3** | I talked less than usual. | 0 | 1 | 2 | 3 |
| **CESDb4** | I felt lonely. | 0 | 1 | 2 | 3 |
| **CESDb5** | People were unfriendly. | 0 | 1 | 2 | 3 |
| **CESDb6** | I enjoyed life. | 0 | 1 | 2 | 3 |
| **CESDb7** | I had crying spells. | 0 | 1 | 2 | 3 |
| **CESDb8** | I felt sad. | 0 | 1 | 2 | 3 |
| **CESDb9** | I felt that people disliked me. | 0 | 1 | 2 | 3 |
| **CESDb10** | I could not “get going.” | 0 | 1 | 2 | 3 |

**GAD** “Over the last 2 weeks, how often have you been bothered by any of the following problems?"

|  |  | **Not at All** | **Several Days** | **More than Half the Days** | **Nearly Every Day** |
| --- | --- | --- | --- | --- | --- |
| **GAD1** | Feeling nervous, anxious or on edge | 0 | 1 | 2 | 3 |
| **GAD2** | Not being able to stop or control worrying | 0 | 1 | 2 | 3 |
| **GAD3** | Worrying too much about different things | 0 | 1 | 2 | 3 |
| **GAD4** | Trouble relaxing | 0 | 1 | 2 | 3 |
| **GAD5** | Being so restless that it is hard to sit still | 0 | 1 | 2 | 3 |
| **GAD6** | Becoming easily annoyed or irritable | 0 | 1 | 2 | 3 |
| **GAD7** | Feeling afraid as if something awful might happen | 0 | 1 | 2 | 3 |

**PHQIntro** “Over the last 2 weeks, how often have you been bothered by any of the following problems?"

|  |  | **Not at All** | **Several Days** | **More than Half the Days** | **Nearly Every Day** |
| --- | --- | --- | --- | --- | --- |
| **PHQ1** | Little interest or pleasure in doing things | 0 | 1 | 2 | 3 |
| **PHQ2** | Feeling down, depressed or hopeless | 0 | 1 | 2 | 3 |

**SelfEsteem** “I feel that I’m a person of worth, at least on an equal plane with others.”

1 Definitely agree

2 Mostly agree

3 Neither agrees nor disagree

4 Mostly disagree

5 Definitely disagree

**IntTransphobia** “How much do you agree or disagree with this statement: I wish I was not transgender or gender nonconforming.”

1 Strongly Disagree

2 Disagree

3 Neither Disagree nor Agree

4 Agree

5 Strongly Agree

**PTSDIntro** “In your life, have you ever had any experience that was so frightening, horrible, or upsetting that, in the past month, you:”

**PTSD1** “Have had nightmares about it or thought about it when you did not want to?”

1 Yes

2 No

**PTSD2** “Tried hard not to think about it or went out of your way to avoid situations that reminded you of it?”

1 Yes

2 No

**PTSD3** “Were constantly on guard, watchful, or easily startled?”

1 Yes

2 No

**PTSD4** “Felt numb or detached from others, activities, or your surroundings?”

1 Yes

2 No

**12. SUBSTANCE USE AND ABUSE ITEMS**

**CRAFFTIntro** “The following questions refer to alcohol and drug use. Remember, your answers are confidential and will not be viewed by clinic staff.”

**CRAFFT1** “Have you ever ridden in a car driven by someone (including yourself) who was “high” or had been using alcohol or drugs?”

1 Yes

2 No

**CRAFFT2** “Do you ever use alcohol or drugs to relax, feel better about yourself, or fit in?”

1 Yes

2 No

**CRAFFT3** “Do you ever use alcohol or drugs while you are by yourself, alone?”

1 Yes

2 No

**CRAFFT4** “Do you ever forget things you did while using alcohol or drugs?”

1 Yes

2 No

**CRAFFT5** “Do your family or friends ever tell you that you should cut down on your drinking or drug use?”

1 Yes

2 No

**CRAFFT6** “Have you ever gotten into trouble while you were using alcohol or drugs?”

1 Yes

2 No

**SubstUseIntro** “Which of the following drugs have you used to get high, feel numb, or feel good in the last 6 months? Please include drugs that were prescribed to you if you used them to get high, feel numb, or feel good. For each drug, please select your frequency of use in the last 6 months.”

|  | **SubstUseIntro1** | Didn’t Use | One time | Once a month or less | Several times per month | About once a week | Several times per week | Daily |
| --- | --- | --- | --- | --- | --- | --- | --- | --- |
|  | In the past 6 months, how often have you used… |  |  |  |  |  |  |  |
| **SubstUse1** | Marijuana (pot) smoked | 0 | 1 | 2 | 3 | 4 | 5 | 6 |
| **SubstUse2** | Marijuana (pot) eaten (brownies or other edibles) | 0 | 1 | 2 | 3 | 4 | 5 | 6 |
| **SubstUse3** | Cocaine (powder) | 0 | 1 | 2 | 3 | 4 | 5 | 6 |
| **SubstUse4** | Crack Cocaine | 0 | 1 | 2 | 3 | 4 | 5 | 6 |
| **SubstUse5** | Club drugs (ecstasy, GHB, ketamine) | 0 | 1 | 2 | 3 | 4 | 5 | 6 |
| **SubstUse6** | Heroin | 0 | 1 | 2 | 3 | 4 | 5 | 6 |
| **SubstUse7** | Methemphetamine (Meth, Tina, Crystal, Speed) | 0 | 1 | 2 | 3 | 4 | 5 | 6 |
| **SubstUse8** | Poppers (amyl nitrate, butyl nitrate) | 0 | 1 | 2 | 3 | 4 | 5 | 6 |
| **SubstUse9** | Hallucinogens (LSD, mushrooms, etc.) | 0 | 1 | 2 | 3 | 4 | 5 | 6 |
| **SubstUse10** | Downers (Valium, Ativan, Xanax, clonopin, etc.) | 0 | 1 | 2 | 3 | 4 | 5 | 6 |
| **SubstUse11** | Painkillers (Oxycontin, Vicodin, Percocet, etc.) | 0 | 1 | 2 | 3 | 4 | 5 | 6 |
| **SubstUse12** | Other Uppers (Adderall, Ritalin, etc.) | 0 | 1 | 2 | 3 | 4 | 5 | 6 |
| **SubstUse13** | Sleep Aids (Nyquil, cough syrup suppressants, other over-the-counter products to help you sleep or numb out, etc.) | 0 | 1 | 2 | 3 | 4 | 5 | 6 |
| **SubstUse14** | Other Drug (please specify): ____________________ | 0 | 1 | 2 | 3 | 4 | 5 | 6 |

**IDU** “Have you injected drugs to get high (IDU) in the last 6 months? (This does not include testosterone injections or hormone injections)”

1 Yes

2 No

**Non-RX** “In the last 6 months, have you taken prescription drugs to get high or feel better that were given to you by a friend but that were not prescribed to you?”

1 Yes, one or twice

2 Yes, a few times

3 Yes, several times

4 Yes, many times

5 No, never

**SubUseTx** “Have you ever in your lifetime been in treatment for alcohol and/or drug abuse?”

1 Yes

2 No

***(Pre-skip to SocialSupportIntro if SubstUse1 AND SubstUse2 both = 0 [no marijuana use])***

**MedMarij** “Do you have a prescription to use marijuana for medicinal purposes?”

1 Yes

2 No

**13. SOCIAL SUPPORT**

**SocialSupportIntro** “The next few questions will be about your relationships with friends and family.”

**FamSupportGender** “In general, how supportive is your family of your gender identity?”

1 Very supportive

2 Somewhat supportive

3 Not very supportive

4 Not at all supportive

5 This does not apply to me

**NumCloseFriends** “How many close friends (people you can confide in) would you say you have?”

**|_____|_____|** Close Friends ***(If NumCloseFriends = 0, skip to FreqSocSupp)***

**NumCloseGenMinFriends** “How many of your close friends (people you can confide in) are transgender or gender nonconforming?”

**|_____|_____|** Close Friends

**FreqSocSupp** “The following questions ask about social support. How often is someone available

…”

|  | 1 None of the time | 2 A little of the time | 3 Some of the time | 4 Most of the time | 5 All of the time |
| --- | --- | --- | --- | --- | --- |
| To help with daily chores if you are sick? |  |  |  |  |  |
| To get together with you for relaxation? |  |  |  |  |  |
| To understand your problems? |  |  |  |  |  |
| To love you and make you feel wanted? |  |  |  |  |  |

**14. SEXUAL RISK & PROTECTION (SECONDARY PREVENTION WORKING GROUP)**

| **SEXINTRO** “This part of the survey will ask you about sex. Sex is a personal issue that can sometimes be sensitive to talk about.  **SEXINTRO1** We understand that people do not all use the same words or names to talk about body parts. This makes it hard for us to ask questions about sex that everyone who is participating in this study can relate to. In this survey, we use the medical words that refer to specific anatomy (parts of the body) —words like penis, anus or butt, and vagina. These may not be the words you use. It is important for this research project that we use words that are clear so that everyone understands what question we are asking. We do not mean to disrespect you in any way.  Let's briefly go over the definitions of some terms so that you understand what is being asked.  **When I say: I mean:**  Penis A flesh penis. This does not include a dildo, toy, or finger.  Vagina A flesh vagina. This does not include a toy, anus, or butt.  Oral Sex When one partner puts her/his mouth on the other person’s penis or vagina.  Vaginal Sex When a penis is put into a vagina.  Receptive Anal Sex When a penis is put into your anus or butt.  Insertive Anal Sex When you put your penis into the anus or butt of your  partner.  Transgender Transgender people have a sex at birth that is different from their current gender identity. For example, a person who was assigned a male sex at birth who identifies their current gender as a woman or female.  Transgender Man A person with a female sex at birth who identifies their current gender as a man, male, or another diverse gender identity (for example, genderqueer).  Transgender Woman A person with a male sex at birth who identifies their current gender as a woman, female, or another diverse gender identity (for example, genderqueer).  The following questions are about times that you had different types of sex because you wanted to, not because you were forced or pressured to have sex. Please answer the following questions and remember to only think about the times you had the different types of sex because you wanted to. Remember that your answers are confidential.  We will ask you about vaginal sex, anal sex, and oral sex. We will also ask you about your partners—including male, female, and transgender partners.” |
| --- |

**TGRecon** “In order to ask you accurate questions about sexual behavior, we will first ask you about your body. We know some transgender people have gender affirming surgery (genital reconstruction such as the creation of a vagina or a penis). Have you had genital reconstruction (i.e., “lower” or “bottom” surgery)?”

1Yes. I have had genital reconstruction

2 No. I have not had genital reconstruction. I have my birth anatomy.

**SexBirthMale6Mos** “During the past 6 months, have you had oral, anal, or vaginal sexual contact with a male partner(s)? By male partners, we mean partners who were assigned a male sex at birth and who identify as male. This does not include transgender partners.”

1 Yes

2 No

**SexBirthFemale6Mos** “During the past 6 months, have you had oral, anal, or vaginal sexual contact with a female partner(s)? By female partners, we mean partners who were assigned a female sex at birth and who identify as female. This does not include transgender partners.”

1 Yes

2 No

**SexTrans6Mos** “During the past 6 months, have you had oral, anal, or vaginal sexual contact with a transgender partner(s)? By transgender partners, we mean partners who were assigned a sex at birth that is different from their current gender identity.”

1 Yes

2 No

**FOR NATAL FEMALES, TRANS MEN NO SURGERY, & TRANS WOMEN POST-SURGERY**

**Note to Programmer:**

If **DemGender=2 AND TGRecon=2**, skip to **MaleSexFemaleIntro.**

If **DemGender=1 AND TGRecon=1**, skip to **MaleSexFemaleIntro**.

If **SexBirthMale6Mos** = 0, skip to **FemaleSexFemaleIntro**.

**SEX WITH NATAL MALES [VAGINA+PENIS]**

**SexFemaleIntro** “Now I am going to ask you about the different types of sex that you have had with males in the past 6 months. By male partners, I mean partners that were assigned a male sex at birth and who identify as male. This does not include transgender partners, which we will ask about later.”

**FemaleSexMale6Mos** “During the past 6 months, how many male partners have you had sexual contact with (oral, anal, or vaginal)?”

**|_____|_____|_____|** Partners (If FemaleSexMale6Mos > 0 AND < 21, skip to FemaleSexMaleHIVPos. If FemaleSexMale6Mos >= 21, skip to FemaleSexMale6MosCheck.)

**FemaleSexMale6MosZero** “You said that you have had no male partners in the past 6 months. Is this correct?”

1 Yes (Skip to FemaleSexFemaleIntro)

2 No (Skip to FemaleSexFemale6Mos)

**FemaleSexMale6MosCheck** “You said that you have had [%FemaleSexMale6Mos%] male partners in the past 6 months. Is this correct?”

1 Yes

2 No (Skip to FemaleSexFemale6Mos)

**FemaleSexMaleHIVPos** “Of these males, how many were known to be HIV positive?”

**|_____|_____|_____|** Partners

**Note to Programmer:** If **FemaleSexMaleHIVPos** = 0, SKIP TO **FemaleSexMaleHIVNegUnk.**

**FemaleSexMaleHIVPosStem** “With your known HIV positive male partners, during the past 6 months:”

**FemalePosMalePerfOralNoCond** “How many times have you performed oral sex WITHOUT a condom?”

**|_____|_____|_____|** Times

**FemalePosMalePerfOralCondom** “How many times have you performed oral sex WITH a condom?”

**|_____|_____|_____|** Times

**FemalePosMaleRcveOralNoCond** “How many times have you received oral sex WITHOUT a barrier (dental dam)?”

**|_____|_____|_____|** Times

**FemalePosMaleRcveOralCond** “How many times have you received oral sex WITH a barrier (dental dam)?”

**|_____|_____|_____|** Times

**FemalePosMaleVagNoCond** “How many times did you have vaginal sex WITHOUT a condom?”

**|_____|_____|_____|** Times

**FemalePosMaleVagCondom** “How many times did you have vaginal sex WITH a condom?”

**|_____|_____|_____|** Times

**FemalePosMaleAnalNoCond** “How many times did you have anal sex WITHOUT a condom?”

**|_____|_____|_____|** Times

**FemalePosMaleAnalCond** “How many times did you have anal sex WITH a condom?”

**|_____|_____|_____|** Times

**FemaleSexMaleHIVNegUnk** “During the past 6 months, how many of your male partners were HIV negative or of unknown HIV status?”

**|_____|_____|_____|** Partners

**Note to programmer:** If **FemaleSexMaleHIVNegUnk**=0, skip to **FemaleSexFemaleIntro**.

**FemaleSexMaleHIVNegStem** “With your HIV negative and HIV unknown status male partners during the past 6 months:”

**FemaleNegMalePerOralNoCo** “How many times have you performed oral sex WITHOUT a condom?”

**|_____|_____|_____|** Times

**FemaleNegMalePerfOralCondom** “How many times have you performed oral sex WITH a condom?”

**|_____|_____|_____|** Times

**FemaleNegMaleRcveOralNoCond** “How many times have you received oral sex WITHOUT a barrier (dental dam)?”

**|_____|_____|_____|** Times

**FemaleNegMaleRcveOralCondom** “How many times have you received oral sex WITH a barrier (dental dam)?”

**|_____|_____|_____|** Times

**FemaleNegMaleVagNoCond** “How many times did you have vaginal sex WITHOUT a condom?”

**|_____|_____|_____|** Times

**FemaleNegMaleVagCondom** “How many times did you have vaginal sex WITH a condom?”

**|_____|_____|_____|** Times

**FemaleNegMaleAnalNoCond** “How many times did you have anal sex WITHOUT a condom?”

**|_____|_____|_____|** Times

**FemaleNegMaleAnalCondom** “How many times did you have anal sex WITH a condom?”

**|_____|_____|_____|** Times

**SEX WITH NATAL FEMALES [VAGINA+VAGINA]**

**Note to Programmer:** If **SexBirthFemale6Mos** = 0, go to **SexTransIntro**.

**FemaleSexFemaleIntro** “Now I am going to ask you about different types of sex that you have had with females in the past 6 months. By female partners, I mean partners that were assigned a female sex at birth and who identify as female. This does not include transgender partners which we will ask about later.”

**FemaleSexFemale6Mos** “During the past 6 months, how many female partners have you had sexual contact with?”

**|_____|_____|_____|** Partners (If FemaleSexFemale6Mos > 0 AND < 21, skip to FemaleSexFemaleHIVPos. If FemaleSexFemale6Mos >= 21, skip to FemaleSexFemale6MosCheck.)

**FemaleSexFemale6MosZero** “You said that you have had no female partners in the past 6 months. Is this correct?”

1 Yes (Skip to SexTransIntro)

2 No (Skip to FemaleSexFemale6Mos)

**FemaleSexFemale6MosCheck** “You said that you have had [%FemaleSexFemale6Mos%] female partners in the past 6 months. Is this correct?”

1 Yes

2 No (Skip to FemaleSexFemale6Mos)

**FemaleSexFemaleHIVPos** “Of these females, how many were known to be HIV positive?”

**|_____|_____|_____|** Partners

If **FemaleSexFemaleHIVPos** = 0, SKIP TO **FemaleSexFemaleHIVNegUnk**

**FemalePosFemalePerfOralNoCond** “With your known HIV positive female partners during the past 6 months:”

**FemalePosFemalePerfOralNoCond** “How many times have you performed oral sex WITHOUT a barrier (dental dam)?”

**|_____|_____|_____|** Times

**FemalePosFemalePerfOralCondom** “How many times have you performed oral sex WITH a barrier (dental dam)?”

**|_____|_____|_____|** Times

**FemalePosFemaleRcveOralNoCond** “How many times have you received oral sex WITHOUT a barrier (dental dam)?”

**|_____|_____|_____|** Times

**FemalePosFemaleRcveOralCondom** “How many times have you received oral sex WITH a barrier (dental dam)?”

**|_____|_____|_____|** Times

**FemalePosFemaleToysWithout** “How many times have you and a partner taken turns with sex toys (for examplefor example, dildos or penetration toys) in your vaginas or butts WITHOUT washing them (or putting fresh condoms on them) in between?”

**|_____|_____|_____|** Times

**FemalePosFemaleToysWith** “How many times have you and a partner took turns with sex toys (for examplefor example, dildos or penetration toys) in your vaginas or butts AND you washed them (or put fresh condoms on them) in between?”

**|_____|_____|_____|** Times

**FemaleSexFemaleHIVNegUnk** “During the past 6 months, how many of your female partners were HIV negative or of unknown HIV status?”

**|_____|_____|_____|** Partners

If **FemaleSexFemaleHIVNegUnk** = 0, SKIP TO **SexTransIntro.**

**SexFemaleHIVNegStem** “With your HIV negative and HIV unknown status female partners during the past 6 months:”

**FemaleNegFemalePerfOralNoCond** “How many times have you performed oral sex WITHOUT a barrier (dental dam)?”

**|_____|_____|_____|** Times

**FemaleNegFemalePerfOralCondom** “How many times have you performed oral sex WITH a barrier (dental dam)?”

**|_____|_____|_____|** Times

**FemaleNegFemaleRcveOralNoCond** “How many times have you received oral sex WITHOUT a barrier (dental dam)?”

**|_____|_____|_____|** Times

**FemaleNegFemaleRcveOralCondom** “How many times have you received oral sex WITH a barrier (dental dam)?”

**|_____|_____|_____|** Times

**FemaleNegFemaleToysWithout** “How many times have you and a partner taken turns with sex toys (for examplefor example, dildos or penetration toys) in your vaginas or butts WITHOUT washing them (or putting fresh condoms on them) in between?”

**|_____|_____|_____|** Times

**FemaleNegFemaleToysWith** “How many times have you and a partner taken turns with sex toys (for examplefor example, dildos or penetration toys) in your vaginas or butts AND you washed them (or put fresh condoms on them) in between?”

**|_____|_____|_____|** Times

**SEX WITH TRANS PARTNERS [VAGINA+…]**

**Note to Programmer:** If **SexTrans6Mos** = 0, go to **LastSex**.

**SexTransIntro** “During the past 6 months, how many transgender partners have you had sexual contact with? By transgender partners, I mean partners that were assigned a sex at birth that is different from their current gender identity.”

**TransSex6MosF** “During the past 6 months, how many transgender partners have you had sexual contact with?”

**|_____|_____|_____|** Partners (If TransSex6MosF > 0 AND < 21, skip to TransWomen6MosF. If TransSex6MosF >= 21, skip to TransSex6MosFCheck.)

**TransSex6MosFZero** “You said that you have had no transgender partners in the past 6 months. Is this correct?”

1 Yes (Skip to LastSex)

2 No (Skip to TransSex6MosF)

**TransSex6MosFCheck** “You said that you have had [%TransSex6MosF%] transgender partners in the past 6 months. Is this correct?”

1 Yes

2 No (Skip to TransSex6MosF)

**TransWoman6MosF** “Of these [%TransSex6MosF%] transgender partners, how many were transgender women, i.e., assigned a male sex at birth?”

**|_____|_____|_____|** Partners **[If “0” skip to TransMan6Mos]**

**TransWomanReconF** “Did any of your partners who were transgender women have genital reconstruction, i.e., “lower” or “bottom” surgery?”
1 Yes, at least one of my partners had genital reconstruction.

2 No, all of my partners had their birth anatomy (penis). **(Skip to TransSexWomanBirthHIVPos)**

**SEX WITH TRANS WOMEN PARTNERS-POST GENITAL RECONSTRUCTION [VAGINA+VAGINA]**

**TransWomanReconHIVPosF** “In the past 6 months, of your transgender woman partners who had gender affirming surgery, how many were known to be HIV positive?”

**|_____|_____|_____|** Partners **[If “0” skip to TransSexWomanReconHIVNegUnk]**

**TransWomanReconHIVPosStemF** “With your HIV positive partners who were transgender women who had gender affirming surgery, during the past 6 months:”

**TransPosWomanReconPerfOralNoCondF** “How many times did you perform oral sex on a transgender woman WITHOUT a barrier (dental dam)?”

**|_____|_____|_____|** Times

**TransPosWomanReconPerfOralCondomF** “How many times did you perform oral sex on a transgender woman WITH a barrier (dental dam)?”

**|_____|_____|_____|** Times

**TransPosWomanReconRcveOralNoCondF** “How many times did you receive oral sex from a transgender woman WITHOUT a barrier (dental dam)?”

**|_____|_____|_____|** Times

**TransPosWomanReconRcveOralCondomF** “How many times did you receive oral sex from a transgender woman WITH a barrier (dental dam)?”

**|_____|_____|_____|** Times

**TransPosWomanReconToysWithout** “How many times did you and a transgender woman take turns with sex toys (for example, dildos or penetration toys) in your vaginas or butts WITHOUT washing them or putting fresh condoms on them in between?”

**|_____|_____|_____|** Times

**TransPosWomanReconToysWith** “How many times did you and a transgender woman take turns with sex toys in your vaginas or butts AND you washed them or put fresh condoms on them in between?”

**|_____|_____|_____|** Times

**TransSexWomanReconHIVNegUnkF** “During the past 6 months, how many of your partners who were transgender women who had gender affirming surgery were HIV negative or of unknown HIV status?”

**|_____|_____|_____|** Partners

If **TransSexWomanReconHIVNegUnk** = 0, SKIP TO **TransWomanBirthAnatomyF**

**TransWomanReconHIVNegUnkStemF** “With your HIV negative and unknown status partners who were transgender women who had gender affirming surgery, during the past 6 months:”

**TransNegWomanReconPerfOralNoCondF** “How many times did you perform oral sex on a transgender woman WITHOUT a barrier (dental dam)?”

**|_____|_____|_____|** Times

**TransNegWomanReconPerfOralCondomF** “How many times did you perform oral sex on a transgender woman WITH a barrier (dental dam)?”

**|_____|_____|_____|** Times

**TransNegWomanReconRcveOralNoCondF** “How many times did you receive oral sex from a transgender woman WITHOUT a barrier (dental dam)?”

**|_____|_____|_____|** Times

**TransNegWomanReconRcveOralCondomF** “How many times did you receive oral sex from a transgender woman WITH a barrier (dental dam)?”

**|_____|_____|_____|** Times

**TransNegWomanReconToysWithout** “How many times did you and a transgender woman take turns with sex toys (for examplefor example, dildos or penetration toys) in your vaginas or butts WITHOUT washing them or putting fresh condoms on them in between?”

**|_____|_____|_____|** Times

**TransNegWomanReconToysWith** “How many times did you and a transgender woman take turns with sex toys in your vaginas or butts AND you washed them or put fresh condoms on them in between?”

**|_____|_____|_____|** Times

**TransWomanBirthAnatomyF** “Did any of your partners who were transgender women have their birth anatomy (penis)?”

1 Yes, at least one of my partners had her birth anatomy (penis)

0 No, all of my partners had genital reconstruction **(Skip to TransMan6MosF)**

**SEX WITH TRANS WOMEN PARTNERS-NO GENITAL RECONSTRUCTION [VAGINA+PENIS]**

**TransSexWomanBirthHIVPosF** “In the past 6 months, of your partners who were transgender women who had their birth anatomy, how many were known to be HIV positive?”

**|_____|_____|_____|** Partners **[If “0” skip to** **TransSexWomanBirthHIVNegUnk]**

**TransWomanBirthHIVPosStemF** “With your HIV positive partners who were transgender women who had their birth anatomy, during the past 6 months:”

**TransPosWomanBirthPerfOralNoCondF** “How many times did you perform oral sex on a transgender woman WITHOUT a condom?”

**|_____|_____|_____|** Times

**TransPosWomanBirthPerfOralCondomF** “How many times did you perform oral sex on a transgender woman WITH a condom?”

**|_____|_____|_____|** Times

**TransPosWomanBirthRcveOralNoCondF** How many times did you receive oral sex from a transgender woman WITHOUT a barrier (dental dam)?”

**|_____|_____|_____|** Times

**TransPosWomanBirthRcveOralCondomF** “How many times did you receive oral sex from a transgender woman WITH a barrier (dental dam)?”

**|_____|_____|_____|** Times

**TransPosWomanBirthVagNoCond** “How many times did you have vaginal sex with a transgender woman WITHOUT a condom?”

**|_____|_____|_____|** Times

**TransPosWomanBirthVagCondom** “How many times did you have vaginal sex with a transgender woman WITH a condom?”

**|_____|_____|_____|** Times

**TransPosWomanBirthAnalNoCond** “How many times did you have anal sex with a transgender woman WITHOUT a condom?”

**|_____|_____|_____|** Times

**TransPosWomanBirthAnalCondom** “How many times did you have anal sex with a transgender woman WITH a condom?”

**|_____|_____|_____|** Times

**TransSexWomanBirthHIVNegUnkF** “During the past 6 months, how many of your partners who were transgender women who had their birth anatomy were HIV negative or of unknown HIV status?”

**|_____|_____|_____|** Partners *[***If “0” skip to TransMan6Mos or other section depending on initial mapping**]

**TransWomanBirthHIVNegStemF** “With your HIV negative and HIV unknown status partners who were transgender women who had their birth anatomy, during the past 6 months:”

**TransNegWomanBirthPerfOralNoCondF** “How many times did you perform oral sex on a transgender woman WITHOUT a condom?”

**|_____|_____|_____|** Times

**TransNegWomanBirthPerfOralCondomF** “How many times did you perform oral sex on a transgender woman WITH a condom?”

**|_____|_____|_____|** Times

**TransNegWomanBirthRcveOralNoCondF** “How many times did you receive oral sex from a transgender woman WITHOUT a barrier, such as a dental dam?”

**|_____|_____|_____|** Times

**TransNegWomanBirthRcveOralCondomF** “How many times did you receive oral sex from a transgender woman WITH a barrier?”

**|_____|_____|_____|** Times

**TransNegWomanBirthVagNoCond** “How many times did you have vaginal sex with a transgender woman WITHOUT a condom?”

**|_____|_____|_____|** Times

**TransNegWomanBirthVagCondom** “How many times did you have vaginal sex with a transgender woman WITH a condom?”

**|_____|_____|_____|** Times

**TransNegWomanBirthAnalNoCond** “How many times did you have anal sex with a transgender woman WITHOUT a condom?”

**|_____|_____|_____|** Times

**TransNegWomanBirthAnalCondom** “How many times did you have anal sex with a transgender woman WITH a condom?”

**|_____|_____|_____|** Times

**SEX WITH TRANS MEN PARTNERS [VAGINA+…]**

**TransMan6MosF** “You told us that you have had sexual contact with [%TransSex6MosF%] transgender partners in the past 6 months. Of these partners, how many were transgender men, that is, assigned a female sex at birth?”

**|_____|_____|_____|** Partners (If TransMan6MosF > 0 AND < 21, skip to TransManReconF. If TransSex6MosF >= 21, skip to TransMan6MosFCheck.)

**TransMan6MosFZero** “You said that you have had no transgender male partners in the past 6 months. Is this correct?”

1 Yes (Skip to LastSex)

2 No (Skip to TransMan6MosF)

**TransMan6MosFCheck** “You said that you have had [%TransSex6MosF%] transgender male partners in the past 6 months. Is this correct?”

1 Yes

2 No (Skip to TransMan6MosF)

**TransManReconF** “We know some transgender people have gender affirming surgery, that is, genital reconstruction such as the creation of a penis. Did any of your partners who are transgender men have genital reconstruction, that is, “lower” or “bottom” surgery?”

1 Yes, at least one of my partners had genital reconstruction

2 No, all of my partners had his birth anatomy (vagina) **(Skip to TransSexManBirthHIVPosF)**

**SEX WITH TRANS MEN PARTNERS-POST GENITAL RECONSTRUCTION [VAGINA+PENIS]**

**TransManReconHIVPosF** “In the past 6 months, of these partners who were transgender men who had gender affirming surgery, how many were known to be HIV positive?”

**|_____|_____|_____|** Partners **[If “0”** **skip to TransManReconHIVNegUnk]**

**TransManReconHIVPosStemF** “With your HIV positive partners who were transgender men who had gender affirming surgery, during the past 6 months:”

**TransPosManReconPerfOralNoCondF** “How many times did you perform oral sex on a transgender man WITHOUT a condom?”

**|_____|_____|_____|** Times

**TransPosManReconPerfOralCondomF** “How many times did you perform oral sex on a transgender man WITH a condom?”

**|_____|_____|_____|** Times

**TransPosManReconRcveOralNoCondF** “How many times did you receive oral sex from a transgender man WITHOUT a barrier (dental dam)?”

**|_____|_____|_____|** Times

**TransPosManReconRcveOralCondomF** “How many times did you receive oral sex from a transgender man WITH a barrier (dental dam)?”

**|_____|_____|_____|** Times

**TransPosManReconVagNoCond** “How many times did you have vaginal sex with a transgender man WITHOUT a condom?”

**|_____|_____|_____|** Times

**TransPosManReconVagCondom** “How many times did you have vaginal sex with a transgender man WITH a condom?”

**|_____|_____|_____|** Times

**TransPosManReconAnalNoCond** “How many times did you have anal sex with a transgender man WITHOUT a condom?”

**|_____|_____|_____|** Times

**TransPosManReconAnalCondom** “How many times did you have anal sex with a transgender man WITH a condom?”

**|_____|_____|_____|** Times

**TransManReconHIVNegUnkF** “During the past 6 months, how many of your partners who were transgender men who had gender affirming surgery were HIV negative or of unknown HIV status?”

**|_____|_____|_____|** Partners **[If “0”** **skip to** **TransManBirthAnatomyF]**

**TransManReconHIVNegStemF** “With your HIV negative and HIV unknown status partners who were transgender men who had gender affirming surgery, during the past 6 months:”

**TransNegManReconPerfOralNoCondF** “How many times did you perform oral sex on a transgender man WITHOUT a condom?”

**|_____|_____|_____|** Times

**TransNegManReconPerfOralCondomF** “How many times did you perform oral sex on a transgender man WITH a condom?”

**|_____|_____|_____|** Times

**TransNegManReconRcveOralNoCondF** “How many times did you receive oral sex from a transgender man WITHOUT a barrier, such as a dental dam?”

**|_____|_____|_____|** Times

**TransNegManReconRcveOralCondomF** “How many times did you receive oral sex from a transgender man WITH a barrier?”

**|_____|_____|_____|** Times

**TransNegManReconVagNoCond** “How many times did you have vaginal sex with a transgender man WITHOUT a condom?”

**|_____|_____|_____|** Times

**TransNegManReconVagCondom** “How many times did you have vaginal sex with a transgender man WITH a condom?”

**|_____|_____|_____|** Times

**TransNegManReconAnalNoCond** “How many times did you have anal sex with a transgender man WITHOUT a condom?”

**|_____|_____|_____|** Times

**TransNegManReconAnalCondom** “How many times did you have anal sex with a transgender man WITH a condom?”

**|_____|_____|_____|** Times

**TransManBirthAnatomyF** “Did any of your partners who were transgender men have their birth anatomy (vagina)?”

1 Yes, at least one of my partners had his birth anatomy (vagina)

0 No, all of my partners had genital reconstruction **[skip to LastSex]**

**SEX WITH TRANS MEN PARTNERS-NO GENITAL RECONSTRUCTION [VAGINA+VAGINA]**

**TransSexManBirthHIVPosF** “In the past 6 months, of your partners who were transgender men who had their birth anatomy, how many were known to be HIV positive?”

**|_____|_____|_____|** Partners  **[If “0”** **skip to** **TransSexManBirthHIVNegUnkF]**

**TransManBirthHIVPosStemF** “With your HIV positive partners who are transgender men who had their birth anatomy, during the past 6 months:”

**TransPosManBirthPerfOralNoCondF** “How many times did you perform oral sex on a transgender man WITHOUT a barrier, such as a dental dam?”

**|_____|_____|_____|** Times

**TransPosManBirthPerfOralCondomF** “How many times did you perform oral sex on a transgender man WITH a barrier?”

**|_____|_____|_____|** Times

**TransPosManBirthRcveOralNoCondF** “How many times did you receive oral sex from a transgender man WITHOUT a barrier?”

**|_____|_____|_____|** Times

**TransPosManBirthRcveOralCondomF** “How many times did you receive oral sex from a transgender man WITH a barrier?”

**|_____|_____|_____|** Times

**TransPosManBirthToysWithout** “How many times did you and a transgender man take turns with sex toys (for examplefor example, dildos or penetration toys) in your vaginas or butts WITHOUT washing them or putting fresh condoms on them in between?”

**|_____|_____|_____|** Times

**TransPosManBirthToysWith** “How many times did you and a transgender man take turns with sex toys (for examplefor example, dildos or penetration toys) in your vaginas or butts AND you washed them or put fresh condoms on them in between?”

**|_____|_____|_____|** Times

**TransSexManBirthHIVNegUnkF** “During the past 6 months, how many of your partners who were transgender men who had their birth anatomy were HIV negative or of unknown HIV status?”

**|_____|_____|_____|** Partners **[If “0”** **skip to LastSex]**

**TransManBirthHIVNegStemF** “With your HIV negative and HIV unknown status partners who were transgender men who had their birth anatomy, during the past 6 months:”

**TransNegManBirthPerfOralCondomF** “How many times did you perform oral sex on a transgender man WITHOUT a barrier, such as a dental dam?”

**|_____|_____|_____|** Times

**TransNegManBirthPerfOralCondomF** “How many times did you perform oral sex on a transgender man WITH a barrier?”

**|_____|_____|_____|** Times

**TransNegManBirthRcveOralNoCondF** “How many times did you receive oral sex from a transgender man WITHOUT a barrier?”

**|_____|_____|_____|** Times

**TransNegManBirthRcveOralCondomF** “How many times did you receive oral sex from a transgender man WITH a barrier?”

**|_____|_____|_____|** Times

**TransNegManBirthToysWithout** “How many times did you and a transgender man take turns with sex toys (for examplefor example, dildos or penetration toys) in your vaginas or butts WITHOUT washing them or putting fresh condoms on them in between?”

**|_____|_____|_____|** Times

**TransNegManBirthToysWith** “How many times did you and a transgender man take turns with sex toys in your vaginas or butts AND you washed them or put fresh condoms on them in between?”

**|_____|_____|_____|** Times

**FOR NATAL MALES, TRANS WOMEN NO SURGERY, & TRANS MEN POST-SURGERY:**

**Note to Programmer:** If **SexBirthFemale6Mos** = 0, go to **SexMaleIntro**.

Ask these questions if:

DemGender = 1 AND TGRecon = 1 OR

DemGender = 2 AND TGRecon = 2

**SEX WITH NATAL FEMALES [PENIS+VAGINA]**

**MaleSexFemaleIntro** “Now I am going to ask you about different types of sex that you have had with females in the past 6 months.”

**MaleSexFemale6Mos** “During the past 6 months, how many female partners have you had oral, anal, or vaginal sexual contact with? By female partners, we mean partners that were assigned a female sex at birth and who identify as female. This does not include transgender partners.”

**|_____|_____|_____|** Partners (If MaleSexFemale6Mos > 0 AND < 21, skip to MaleSexFemaleHIVPos. If MaleSexFemale6Mos >= 21, skip to MaleSexFemale6MosCheck.)

**MaleSexFemale6MosZero** “You said that you have had no female partners in the past 6 months. Is this correct?”

1 Yes (Skip to SexMaleIntro)

2 No (Skip to MaleSexFemale6Mos)

**MaleSexFemale6MosCheck** “You said that you have had [%MaleSexFemale6Mos%] female partners in the past 6 months. Is this correct?”

1 Yes

2 No (MaleSexFemale6Mos)

**MaleSexFemaleHIVPos** “Of these females, how many were known to be HIV positive?”

**|_____|_____|_____|** Partners **[If 0, skip to MaleSexFemaleHIVNegUnk]**

**MaleSexFemaleHIVPosStem** “With your known HIV positive female partners during the past 6 months:”

**MalePosFemalePerfOralNoCond** “How many times have you performed oral sex WITHOUT a barrier (dental dam)?”

**|_____|_____|_____|** Times

**MalePosFemalePerfOralCondom** “How many times have you performed oral sex WITH a barrier (dental dam)?”

**|_____|_____|_____|** Times

**MalePosFemaleRcveOralNoCond** “How many times did you receive oral sex WITHOUT a condom?”

**|_____|_____|_____|** Times

**MalePosFemaleRcveOralCondom** “How many times did you receive oral sex WITH a condom?”

**|_____|_____|_____|** Times

**MalePosFemaleVagNoCond** “How many times did you have vaginal sex WITHOUT a condom?”

**|_____|_____|_____|** Times

**MalePosFemaleVagCondom** “How many times did you have vaginal sex WITH a condom?”

**|_____|_____|_____|** Times

**MalePosFemaleAnalNoCond** “How many times did you have anal sex WITHOUT a condom?”

**|_____|_____|_____|** Times

**MalePosFemaleAnalCondom** “How many times did you have anal sex WITH a condom?”

**|_____|_____|_____|** Times

**MaleSexFemaleHIVNegUnk** “During the past 6 months, how many of your female partners were HIV negative or of unknown HIV status?”

**|_____|_____|_____|** Partners

If **MaleSexFemaleHIVNegUnk** = 0, SKIP TO **SexMaleIntro**

“With your HIV negative and HIV unknown status female partners during the past 6 months:”

**MaleNegFemalePerfOralNoCond** “How many times have you performed oral sex WITHOUT a barrier (dental dam)?”

**|_____|_____|_____|** Times

**MaleNegFemalePerfOralCondom** “How many times have you performed oral sex WITH a barrier (dental dam)?”

**|_____|_____|_____|** Times

**MaleNegFemaleRcveOralNoCond** “How many times did you receive oral sex WITHOUT a condom?”

**|_____|_____|_____|** Times

**MaleNegFemaleRcveOralCondom** “How many times did you receive oral sex WITH a condom?”

**|_____|_____|_____|** Times

**MaleNegFemaleVagNoCond** “How many times did you have vaginal sex WITHOUT a condom?”

**|_____|_____|_____|** Times

**MaleNegFemaleVagCondom** “How many times did you have vaginal sex WITH a condom?”

**|_____|_____|_____|** Times

**MaleNegFemaleAnalNoCond** “How many times did you have anal sex WITHOUT a condom?”

**|_____|_____|_____|** Times

**MaleNegFemaleAnalCondom** “How many times did you have anal sex WITH a condom?”

**|_____|_____|_____|** Times

**SEX WITH NATAL MALES [PENIS+PENIS]**

**Note to Programmer:** If **SexBirthMale6Mos** = 0, SKIP TO **TransSex6MosM.**

**SexMaleIntro** “Now I am going to ask you about the different types of sex that you have had with males in the past 6 months. By male partners, we mean partners that were assigned a male sex at birth and who identify as male. This does not include transgender partners which we will ask about later.”

**MaleSexMale6Mos** “During the past 6 months, how many male partners have you had sexual contact (oral or anal) with?”

**|_____|_____|_____|** Partners (If MaleSexMale6Mos > 0 AND < 21, skip to MaleSexMaleHIVPos. If MaleSexMale6Mos >= 21, skip to MaleSexMale6MosCheck.)

**MaleSexMale6MosZero** “You said that you have had no male partners in the past 6 months. Is this correct?”

1 Yes (Skip to TransSex6MosM)

2 No (Skip to MaleSexMale6Mos)

**MaleSexMale6MosCheck** “You said that you have had [%MaleSexFemale6Mos%] male partners in the past 6 months. Is this correct?”

1 Yes

2 No (MaleSexMale6Mos)

**MaleSexMaleHIVPos** “Of these males, how many were known to be HIV positive?”

**|_____|_____|_____|** Partners **[**If **MaleSexMaleHIVPos** = 0, SKIP TO **MaleSexMaleHIVNegUnk.]**

**MaleSexMaleHIVPosStem** “With your known HIV positive male partners during the past 6 months:”

**MalePosMalePerfOralNoCond** “How many times have you performed oral sex WITHOUT a condom?”

**|_____|_____|_____|** Times

**MalePosMalePerfOralCondom** “How many times have you performed oral sex WITH a condom?”

**|_____|_____|_____|** Times

**MalePosMaleRcveOralNoCond** “How many times did you receive oral sex WITHOUT a condom?”

**|_____|_____|_____|** Times

**MalePosMaleRcveOralCondom** “How many times did you receive oral sex WITH a condom?”

**|_____|_____|_____|** Times

**MalePosMaleInsAnalNoCond** How many times did you have insertive anal sex WITHOUT a condom?”

**|_____|_____|_____|** Times

**MalePosMaleInsAnalCondom** How many times did you have insertive anal sex WITH a condom?”

**|_____|_____|_____|** Times

**MalePosMaleRecAnalNoCond** How many times did you have receptive anal sex WITHOUT a condom?”

**|_____|_____|_____|** Times

**MalePosMaleRecAnalCondom** “How many times did you have receptive anal sex WITH a condom?”

**|_____|_____|_____|** Times

**MaleSexMaleHIVNegUnk** “During the past 6 months, how many of your male partners were HIV negative or of unknown HIV status?”

**|_____|_____|_____|** Partners [If **MaleSexMaleHIVNegUnk** = 0, SKIP TO **TransSex6MosM**.]

**MaleSexMaleHIVNegStem** “With your HIV negative and HIV unknown status male partners during the past 6 months:”

**MaleNegMalePerfOralNoCond** “How many times have you performed oral sex WITHOUT a condom?”

**|_____|_____|_____|** Times

**MaleNegMalePerfOralCondom** “How many times have you performed oral sex WITH a condom?”

**|_____|_____|_____|** Times

**MaleNegMaleRcveOralNoCond** “How many times did you receive oral sex WITHOUT a condom?”

**|_____|_____|_____|** Times

**MaleNegMaleRcveOralCondom** “How many times did you receive oral sex WITH a condom?”

**|_____|_____|_____|** Times

**MaleNegMaleInsAnalNoCond** “How many times did you have insertive anal sex WITHOUT a condom?”

**|_____|_____|_____|** Times

**MaleNegMaleInsAnalCondom** “How many times did you have insertive anal sex WITH a condom?”

**|_____|_____|_____|** Times

**MaleNegMaleRecAnalNoCond** “How many times did you have receptive anal sex WITHOUT a condom?”

**|_____|_____|_____|** Times

**MaleNegMaleRecAnalCondom** “How many times did you have receptive anal sex WITH a condom?”

**|_____|_____|_____|** Times

**SEX WITH TRANSGENDER PARTNERS [PENIS+…]**

**Note to Programmer:** If **SexTrans6Mos** = 0, SKIP TO **LastSex**.

**TransSex6MosM** “During the past 6 months, how many transgender partners have you had sexual contact (oral, anal, or vaginal) with?”

**|_____|_____|_____|** Partners (If TransSex6MosM > 0 AND < 21, skip to TransWoman6MosM. If TransSex6MosM >= 21, skip to TransSex6MosMCheck.)

**TransSex6MosMZero** “You said that you have had no transgender partners in the past 6 months. Is this correct?”

1 Yes (Skip to LastSex)

2 No (Skip to TransSex6MosM)

**TransSex6MosMCheck** “You said that you have had [%TransSex6MosM%] transgender partners in the past 6 months. Is this correct?”

1 Yes

2 No (TransSex6MosM)

**TransWoman6MosM** “Of these [%TransSex6MosM%] transgender partners, how many were transgender women, i.e., assigned a male sex at birth?”

**|_____|_____|_____|** Partners **[If “0”** **skip to TransMan6MosM]**

**TransWomanReconM** “We know some transgender people have gender affirming surgery, i.e., genital reconstruction such as the creation of a vagina. Did any of your transgender female partners have genital reconstruction, i.e., “lower” or “bottom” surgery?”

1 Yes, at least one of my partners had genital reconstruction

2 No, all of my partners had her birth anatomy (penis) **[skip to TransSexWomanBirthHIVPosM]**

**SEX WITH TRANS WOMEN PARTNERS-POST GENITAL RECONSTRUCTION [PENIS+VAGINA]**

**TransWomanReconHIVPosM** “In the past 6 months, of your partners who were transgender women who had gender affirming surgery, how many were known to be HIV positive?”

**|_____|_____|_____|** Partners **[If “0”** **skip to** **TransSexWomanReconHIVNegUnkM]**

**TransPosWomanReconHIVPosStemM** “With your HIV positive transgender woman partners who had gender affirming surgery, during the past 6 months:”

**TransPosWomanReconPerfOralNoCondM** “How many times did you perform oral sex WITHOUT a barrier (dental dam)?”

**|_____|_____|_____|** Times

**TransPosWomanReconPerfOralCondomM** “How many times did you perform oral sex WITH a barrier (dental dam)?”

**|_____|_____|_____|** Times

**TransPosWomanReconRcveOralNoCondM** “How many times did you receive oral sex WITHOUT a condom?”

**|_____|_____|_____|** Times

**TransPosWomanReconRcveOralCondomM** “How many times did you receive oral sex WITH a condom?”

**|_____|_____|_____|** Times”

**TransPosWomanReconVagNoCond** “How many times did you have vaginal sex with a woman WITHOUT a condom?”

**|_____|_____|_____|** Times

**TransPosWomanReconVagCondom** “How many times did you have vaginal sex WITH a condom?”

**|_____|_____|_____|** Times

**TransPosWomanReconAnalNoCond** “How many times did you have anal sex WITHOUT a condom?”

**|_____|_____|_____|** Times

**TransPosWomanReconAnalCondom** “How many times did you have anal sex WITH a condom?”

**|_____|_____|_____|** Times

**TransSexWomanReconHIVNegUnkM** “During the past 6 months, how many of your transgender woman partners who had gender affirming surgery were HIV negative or of unknown HIV status?”

**|_____|_____|_____|** Partners **[If “0”** **skip to** **TransWomanBirthAnatomyM]**

**TransWomanReconHIVNegUnkStemM** “With your HIV negative and HIV unknown status transgender woman partners who had gender affirming surgery, during the past 6 months:”

**TransNegWomanReconPerfOralNoCondM** “How many times did you perform oral sex WITHOUT a barrier (dental dam)?”

**|_____|_____|_____|** Times

**TransNegWomanReconPerfOralCondomM** “How many times did you perform oral sex WITH a barrier (dental dam)?”

**|_____|_____|_____|** Times

**TransNegWomanReconRcveOralNoCondM** “How many times did you receive oral sex WITHOUT a condom?”

**|_____|_____|_____|** Times

**TransNegWomanReconRcveOralCondomM** “How many times did you receive oral sex WITH a condom?”

**|_____|_____|_____|** Times

**TransNegWomanReconRcveVagNoCond** “How many times did you have vaginal sex WITHOUT a condom?”

**|_____|_____|_____|** Times

**TransNegWomanReconRcveVagCondom** “How many times did you have vaginal sex WITH a condom?”

**|_____|_____|_____|** Times

**TransNegWomanReconAnalNoCond** “How many times did you have anal sex WITHOUT a condom?”

**|_____|_____|_____|** Times

**TransNegWomanReconAnalCondom** “How many times did you have anal sex WITH a condom?”

**|_____|_____|_____|** Times

**TransWomanBirthAnatomyM** “Did any of your partners who were transgender women have their birth anatomy (penis)?”

1 Yes, at least one of my partners had her birth anatomy (penis)

0 No, all of my partners had genital reconstruction **[SKIP TO TransMan6MosM]**

**SEX WITH TRANS WOMEN PARTNERS-NO GENITAL RECONSTRUCTION [PENIS+PENIS]**

**TransSexWomanBirthHIVPosM** “In the past 6 months, of your transgender woman partners who had their birth anatomy, how many were known to be HIV positive?”

**|_____|_____|_____|** Partners **[If “0”** **skip to** **TransSexWomanBirthHIVNegUnkM]**

**TransWomanBirthHIVPosStemM** “With your HIV positive transgender woman partners who had their birth anatomy, during the past 6 months:”

**TransPosWomanBirthPerfOralNoCondM** “How many times did you perform oral sex WITHOUT a condom?”

**|_____|_____|_____|** Times

**TransPosWomanBirthPerfOralCondomM** “How many times did you perform oral sex WITH a condom?”

**|_____|_____|_____|** Times

**TransPosWomanBirthRcveOralNoCondM** “How many times did you receive oral sex WITHOUT a condom?”

**|_____|_____|_____|** Times

**TransPosWomanBirthRcveOralCondomM** “How many times did you receive oral sex WITH a condom?”

**|_____|_____|_____|** Times

**TransPosWomanBirthInsAnalNoCond** “How many times did you have insertive anal sex WITHOUT a condom?”  **|_____|_____|_____|** Times

**TransPosWomanBirthInsAnalCondom** “How many times did you have insertive anal sex WITH a condom?”

**|_____|_____|_____|** Times

**TransPosWomanBirthRecAnalNoCond** “How many times did you have receptive anal sex WITHOUT a condom?”

**|_____|_____|_____|** Times

**TransPosWomanBirthRecAnalCondom** “How many times did you have receptive anal sex WITH a condom?”

**|_____|_____|_____|** Times

**TransSexWomanBirthHIVNegUnkM** “During the past 6 months, how many of your transgender woman partners who had their birth anatomy were HIV negative or of unknown HIV status?”

**|_____|_____|_____|** Partners **[If “0”** **skip to** **TransMan6MosM**]

**TransWomanBirthHIVNegStemM** “With your HIV negative and HIV unknown status transgender woman partners who had their birth anatomy, during the past 6 months:”

**TransNegWomanBirthPerfOralNoCondM** “How many times did you perform oral sex WITHOUT a condom?”

**|_____|_____|_____|** Times

**TransNegWomanBirthPerfOralCondomM** “How many times did you perform oral sex WITH a condom?”

**|_____|_____|_____|** Times

**TransNegWomanBirthRcveOralNoCondM** “How many times did you receive oral sex WITHOUT a condom?”

**|_____|_____|_____|** Times

**TransNegWomanBirthRcveOralCondomM** “How many times did you receive oral sex WITH a condom?”

**|_____|_____|_____|** Times

**TransNegWomanBirthInsAnalNoCond** “How many times did you have insertive anal sex WITHOUT a condom?”

**|_____|_____|_____|** Times

**TransNegWomanBirthInsAnalCondom** “How many times did you have insertive anal sex WITH a condom?”

**|_____|_____|_____|** Times

**TransNegWomanBirthRecAnalNoCond** “How many times did you have receptive anal sex WITHOUT a condom?”

**|_____|_____|_____|** Times

**TransNegWomanBirthRecAnalCondom** “How many times did you have receptive anal sex WITH a condom?”

**|_____|_____|_____|** Times

**SEX WITH TRANSMEN PARTNERS [VAGINA+…]**

**TransMan6MosM** “You told us that you have had sexual contact with [%TransSex6MosM%] transgender partners in the past 6 months. Of these transgender partners, how many were transgender men, i.e., assigned a female sex at birth?”

**|_____|_____|_____|** Partners (If TransMan6MosM > 0 AND < 21, skip to TransManReconM. If TransMan6MosM >= 21, skip to TransMan6MosMCheck.)

**TransMan6MosMZero** “You said that you have had no transgender male partners in the past 6 months. Is this correct?”

1 Yes (Skip to LastSex)

2 No (Skip to TransMan6MosM)

**TransMan6MosMCheck** “You said that you have had [%TransMan6MosM%] transgender male partners in the past 6 months. Is this correct?”

1 Yes

2 No (Skip to TransMan6MosM)

**TransManReconM** “We know some transgender people have gender affirming surgery, that is, genital reconstruction such as the creation of a penis. Did any of your partners who are transgender men have genital reconstruction, that is, “lower” or “bottom” surgery?”

1 “Yes, at least one of my partners had genital reconstruction”

2 “No, all of my partners had his birth anatomy (vagina)” **[skip to TransSexManBirthHIVPosM]**

**SEX WITH TRANS MEN PARTNERS-POST GENITAL RECONSTRUCTION [PENIS+PENIS]**

**TransManReconHIVPosM** “In the past 6 months, of your partners who were transgender men who had gender affirming surgery, how many were known to be HIV positive?”

**|_____|_____|_____|** Partners  **[If 0,** **skip to** **TransManReconHIVNegUnkM]**

**TransManReconHIVPosStemM** “With your HIV positive transgender men partners who had gender affirming surgery, during the past 6 months:”

**TransPosManReconPerfOralNoCondM** “How many times did you perform oral sex WITHOUT a condom?”

**|_____|_____|_____|** Times

**TransPosManReconPerfOralCondomM** “How many times did you perform oral sex WITH a condom?”

**|_____|_____|_____|** Times

**TransPosManReconRcveOralNoCondM** “How many times did you receive oral sex WITHOUT a condom?”

**|_____|_____|_____|** Times

**TransPosManReconRcveOralCondomM** “How many times did you receive oral sex from a transgender man WITH a condom?”

**|_____|_____|_____|** Times

**TransPosManReconInsAnalNoCond** “How many times did you have insertive anal sex WITHOUT a condom?”

**|_____|_____|_____|** Times

**TransPosManReconInsAnalCondom** “How many times did you have insertive anal sex WITH a condom?”

**|_____|_____|_____|** Times

**TransPosManReconRecAnalNoCond** “How many times did you have receptive anal sex WITHOUT a condom?”

**|_____|_____|_____|** Times

**TransPosManReconRecAnalCondom** “How many times did you have receptive anal sex WITH a condom?”

**|_____|_____|_____|** Times

**TransManReconHIVNegUnkM** “During the past 6 months, how many of your transgender men partners who had gender affirming surgery were HIV negative or of unknown HIV status?”

**|_____|_____|_____|** Partners  **[If “0”** **skip to TransManBirthAnatomyM]**

**TransManReconHIVNegStemM** “With your HIV negative and HIV unknown status transgender men partners had gender affirming surgery, during the past 6 months:”

**TransNegManReconPerfOralNoCondM** “How many times did you perform oral sex WITHOUT a condom?

**|_____|_____|_____|** Times

**TransNegManReconPerfOralCondomM** “How many times did you perform oral sex WITH a condom?”

**|_____|_____|_____|** Times

**TransNegManReconRcveOralNoCondM** “How many times did you receive oral sex WITHOUT a condom?”

**|_____|_____|_____|** Times

**TransNegManReconRcveOralCondomM** “How many times did you receive oral sex WITH a condom?”

**|_____|_____|_____|** Times

**TransNegManReconInsAnalNoCond** “How many times did you have insertive anal sex WITHOUT a condom?”

**|_____|_____|_____|** Times

**TransNegManReconInsAnalCondom** “How many times did you have insertive anal sex WITH a condom?”

**|_____|_____|_____|** Times

**TransNegManReconRecAnalNoCond** “How many times did you have receptive anal sex with a transgender man WITHOUT a condom?”

**|_____|_____|_____|** Times

**TransNegManReconRecAnalCondom** “How many times did you have receptive anal sex WITH a condom?”

**|_____|_____|_____|** Times

**TransManBirthAnatomyM** “Did any of your partners who were transgender men have their birth anatomy (vagina)?”

1 “Yes, at least one of my partners had his birth anatomy (vagina)”

0 “No, all of my transgender male partners had genital reconstruction” [**Skip to LastSex]**

**SEX WITH TRANS MEN PARTNERS-NO GENITAL RECONSTRUCTION [PENIS+VAGINA]**

**TransSexManBirthHIVPosM** “In the past 6 months, of your transgender men partners who had their birth anatomy, how many were known to be HIV positive?”

**|_____|_____|_____|** Partners **[If “0”** **skip to TransSexManBirthHIVNegUnkM]**

**TransManBirthHIVPosStemM** “With your HIV positive transgender men partners who had their birth anatomy, during the past 6 months:”

**TransPosManBirthPerfOralNoCondF** “How many times did you perform oral sex WITHOUT a barrier (dental dam)?”

**|_____|_____|_____|** Times

**TransPosManBirthPerfOralCondomM** “How many times did you perform oral sex WITH a barrier (dental dam)?”

**|_____|_____|_____|** Times

**TransPosManBirthRcveOralNoCondM** “How many times did you receive oral sex WITHOUT a condom?”

**|_____|_____|_____|** Times

**TransPosManBirthRcveOralCondomM** “How many times did you receive oral sex WITH a condom?”

**|_____|_____|_____|** Times

**TransPosManBirthVagNoCond** “How many times did you have vaginal sex WITHOUT a condom?”

**|_____|_____|_____|** Times

**TransPosManBirthVagCondom** “How many times did you have vaginal sex WITH a condom?”

**|_____|_____|_____|** Times

**TransPosManBirthAnalNoCond** “How many times did you have anal sex WITHOUT a condom? “

**|_____|_____|_____|** Times

**TransPosManBirthAnalCondom** “How many times did you have anal sex WITH a condom?”

**|_____|_____|_____|** Times

**TransSexManBirthHIVNegUnkM “**During the past 6 months, how many of your transgender men partners who had their birth anatomy were HIV negative or of unknown HIV status?”

**|_____|_____|_____|** Partners **[If “0”** **skip to LastSex]**

**TransManBirthHIVNegStemM** “With your HIV negative and HIV unknown status transgender men partners who had their birth anatomy, during the past 6 months:”

**TransNegManBirthPerfOralCondomM** “How many times did you perform oral sex WITHOUT a barrier (dental dam)?”

**|_____|_____|_____|** Times

**TransNegManBirthPerfOralCondomM** “How many times did you perform oral sex WITH a barrier (dental dam)?”

**|_____|_____|_____|** Times

**TransNegManBirthRcveOralNoCondM** “How many times did you receive oral sex WITHOUT a condom?”

**|_____|_____|_____|** Times

**TransNegManBirthRcveOralCondomM** “How many times did you receive oral sex WITH a condom?”

**|_____|_____|_____|** Times

**TransNegManBirthVagNoCond** “How many times did you have vaginal sex WITHOUT a condom?”

**|_____|_____|_____|** Times

**TransNegManBirthVagCondom** “How many times did you have vaginal sex WITH a condom?”

**|_____|_____|_____|** Times

**TransNegManBirthAnalNoCond** “How many times did you have anal sex WITHOUT a condom?”

**|_____|_____|_____|** Times

**TransNegManBirthAnalCondom** “How many times did you have anal sex WITH a condom?”

**|_____|_____|_____|** Times

**LastSex:** “You have reached the end of the current group of questions. Press the NEXT button to go to the next group of questions or press the BACK button to check your previous answers. Once you press the NEXT button, you will no longer be able to return to these questions.”

**15. DRUGS/ALCOHOL AND SEX**

***Pre-skip to DiscrimYear if SexBirthMale6Mos = 0 AND SexBirthFemale6Mos = 0 AND SexTrans6Mos = 0***

**AlcoholSexIntro** “The next questions ask about times when you have or have not used condoms with either male, female, or transgender partners.”

**AlcoholSex6Mos** “In the past 6 months, after drinking alcohol, did you have vaginal or anal sex without a condom even though you had intended to use a condom?”

1 Yes

2 No ***(Skip to MarijuanaSex6Mos)***

***Preskip AlcoholSex6MosPos if:***

***FemaleSexMaleHIVPos < 1 AND FemaleSexFemaleHIVPos < 1 AND TransWomanReconHIVPosF < 1 AND TransSexWoBirthHIVPosF < 1 AND TransManReconHIVPosF < 1 AND TransSexManBirHIVPosF < 1 AND MaleSexFemaleHIVPos < 1 AND MaleSexMaleHIVPos < 1 AND TransWomanReconHIVPosM < 1 AND TransWomanBirthHIVPosM < 1 AND TransManReconHIVPosM < 1 AND TransSexManBirHIVPosM < 1***

**AlcoholSex6MosPos** “How often has this happened in the past 6 months with an HIV positive partner?”

**|_____|_____|_____|** Times

***Preskip AlcoholSex6MosNeg if:***

***FemaleSexMaleHIVNegUnk <1 AND FemaleSexFemHIVNegUnk <1 AND TransWomanReconHIVPosF <1 AND TransSexWoRecoHIVNegUnkF <1 AND TransSexWoBirHIVNegUnkF <1 AND TransManReconHIVNegUnkF <1 AND TransSexManBirHIVNegUnkF <1 AND MaleSexFemHIVNegUnk <1 AND MaleSexMaleHIVNegUnk <1 AND TransSexWoRecHIVNegUnkM <1 AND TransSexWoBirthHIVNegUnM <1 AND TransManReconHIVNegUnkM <1 AND TransSexManBirHIVNegUM <1***

**AlcoholSex6MosNeg** “How often has this happened in the past 6 months with an HIV negative or unknown partner?”

**|_____|_____|_____|** Times

***Ask MarijuanaSex6Mos if SubstUse1 (smoked marijuana past 6 months) > 0 or if SubstUse2 (eaten marijuana past six months) > 0. Otherwise go to OtherDrugSex6Mos.***

**MarijuanaSex6Mos** “In the past 6 months, after smoking marijuana, did you have vaginal or anal sex without a condom even though you had intended to use a condom?”

1 Yes

0 No ***(Skip to OtherDrugsSex6Mos)***

***Preskip MarijuanaSex6MosPos if:***

***FemaleSexMaleHIVPos < 1 AND FemaleSexFemaleHIVPos < 1 AND TransWomanReconHIVPosF < 1 AND TransSexWoBirthHIVPosF < 1 AND TransManReconHIVPosF < 1 AND TransSexManBirHIVPosF < 1 AND MaleSexFemaleHIVPos < 1 AND MaleSexMaleHIVPos < 1 AND TransWomanReconHIVPosM < 1 AND TransWomanBirthHIVPosM < 1 AND TransManReconHIVPosM < 1 AND TransSexManBirHIVPosM < 1*****MarijuanaSex6MosPos** “How often has this happened in the past 6 months with an HIV positive partner?”

**|_____|_____|_____|** Times

***Preskip MarijuanaSex6MosNeg if:***

***FemaleSexMaleHIVNegUnk <1 AND FemaleSexFemHIVNegUnk <1 AND TransWomanReconHIVPosF <1 AND TransSexWoRecoHIVNegUnkF <1 AND TransSexWoBirHIVNegUnkF <1 AND TransManReconHIVNegUnkF <1 AND TransSexManBirHIVNegUnkF <1 AND MaleSexFemHIVNegUnk <1 AND MaleSexMaleHIVNegUnk <1 AND TransSexWoRecHIVNegUnkM <1 AND TransSexWoBirthHIVNegUnM <1 AND TransManReconHIVNegUnkM <1 AND TransSexManBirHIVNegUM <1***

**MarijuanaSex6MosNeg** “How often has this happened in the past 6 months with an HIV negative or unknown partner?”

**|_____|_____|_____|** Times

***Ask OtherDrugsSex6Mos only if SubstUse3, SubstUse4, SubstUse5, SubstUse6, SubstUse7, SubstUse8, SubstUse9, SubstUse10, SubstUse11, SubstUse12, SubstUse13, OR SubstUse14 > 0. Else go to GenderAffirmSex.***

**OtherDrugsSex6Mos** “In the past 6 months, after using other drugs besides alcohol and marijuana, did you have vaginal or anal sex without a condom even though you had intended to use a condom?”

1 Yes

0 No ***(Skip to PronounSex)***

***Preskip OtherDrugsSex6MosPos if:***

***FemaleSexMaleHIVPos < 1 AND FemaleSexFemaleHIVPos < 1 AND TransWomanReconHIVPosF < 1 AND TransSexWoBirthHIVPosF < 1 AND TransManReconHIVPosF < 1 AND TransSexManBirHIVPosF < 1 AND MaleSexFemaleHIVPos < 1 AND MaleSexMaleHIVPos < 1 AND TransWomanReconHIVPosM < 1 AND TransWomanBirthHIVPosM < 1 AND TransManReconHIVPosM < 1 AND TransSexManBirHIVPosM < 1***

**OtherDrugsSex6MosPos** “How often has this happened in the past 6 months with an HIV positive partner?”

**|_____|_____|_____|** Times

***Preskip OtherDrugsSex6MosNeg if:***

***FemaleSexMaleHIVNegUnk <1 AND FemaleSexFemHIVNegUnk <1 AND TransWomanReconHIVPosF <1 AND TransSexWoRecoHIVNegUnkF <1 AND TransSexWoBirHIVNegUnkF <1 AND TransManReconHIVNegUnkF <1 AND TransSexManBirHIVNegUnkF <1 AND MaleSexFemHIVNegUnk <1 AND MaleSexMaleHIVNegUnk <1 AND TransSexWoRecHIVNegUnkM <1 AND TransSexWoBirthHIVNegUnM <1 AND TransManReconHIVNegUnkM <1 AND TransSexManBirHIVNegUM <1* OtherDrugsSex6MosNeg** “How often has this happened in the past 6 months with an HIV negative or unknown partner?”

**|_____|_____|_____|** Times

**16. GENDER AFFIRMATION IN SEXUAL CONTEXT**

***Ask following four questions only if responses to any of the following = 1: SexBirthMale6Mos, SexBirthFemale6Mos, OR SexTrans6Mos. Else skip to DiscrimIntro.***

**GenderAffirmSex** “In the past 6 months, how often have you experienced the following things?”

In the past six months, how often have you…

**GenderAffirmSex1** “… been mispronouned/misgendered during or after sex by a sexual partner? (For example, referred to as “he” when your pronoun is “she”)”

1 Never

2 Occasionally

3 Sometimes

4 Most of the time

5 Always

**GenderAffirmSex2** “… had sex with someone and NOT disclosed your transgender or gender nonconforming status?”

1 Never

2 Occasionally

3 Sometimes

4 Most of the time

5 Always

**GenderAffirmSex3** “… felt pressured to do things sexually you may not have wanted to, because the person you were having sex with validated your gender identity or gender presentation?”

1 Never

2 Occasionally

3 Sometimes

4 Most of the time

5 Always

**GenderAffirmSex4** “… did you have vaginal or anal sex without a condom because it felt good to have your gender identity or gender presentation validated by the person you were having sex with?”

1 Never

2 Occasionally

3 Sometimes

4 Most of the time

5 Always

**17. Experiences of Discrimination**

**DiscrimYear** “This next section asks about experiences that some people have as they go about their daily lives. Please indicate how often you have experienced the following situations in the past year.”

In the past year, have you…

| [**DiscrimYear1**] been treated with less courtesy than others? | Never | Rarely | Sometimes | Often | Very often |
| --- | --- | --- | --- | --- | --- |
| [**DiscrimYear2**] been treated with less respect than others? |  |  |  |  |  |
| [**DiscrimYear3**] received poorer services than others in restaurants or stores? |  |  |  |  |  |
| [**DiscrimYear4**] experienced people treating you as if you’re not smart? |  |  |  |  |  |
| [**DiscrimYear5**] experienced people acting like they were afraid of you? |  |  |  |  |  |
| [**DiscrimYear6**] experienced people acting like you were dishonest? |  |  |  |  |  |
| [**DiscrimYear7**] experienced people acting like they were better than you? |  |  |  |  |  |
| [**DiscrimYear8**] been called names or insulted? |  |  |  |  |  |
| [**DiscrimYear9**] been threatened or harassed? |  |  |  |  |  |

***If DiscrimYear1-9 all = 1 Never, skip to DiscrimMonth***

**DiscrimReasonYear** “In the past year, do you think your experience(s) of being treated with less courtesy than others was related to your…. (Check all that apply)”

1. Age
2. Sex
3. Race
4. Ethnicity
5. Nationality
6. Religion
7. Income Level/Social Class
8. HIV Status
9. Weight
10. Sexual Orientation
11. Gender Identity
12. Gender Expression (how masculine or feminine you appear, i.e., how you walk, talk, or dress)
13. Physical Disability
14. Another Reason (Please Specify: ____________)

**DiscrimMonth** “Now we would like to know how often you have experienced these same situations in the past month.”

In the past 30 days, have you…

| [**DiscrimMonth1**] been treated with less courtesy than others? | Never | Rarely | Sometimes | Often | Very often |
| --- | --- | --- | --- | --- | --- |
| [**DiscrimMonth2**] been treated with less respect than others? |  |  |  |  |  |
| [**DiscrimMonth3**] received poorer services than others in restaurants or stores? |  |  |  |  |  |
| [**DiscrimMonth4**] experienced people treating you as if you’re not smart? |  |  |  |  |  |
| [**DiscrimMonth5**] experienced people acting like they were afraid of you? |  |  |  |  |  |
| [**DiscrimMonth6**] experienced people acting like you were dishonest? |  |  |  |  |  |
| [**DiscrimMonth7**] experienced people acting like they were better than you? |  |  |  |  |  |
| [**DiscrimMonth8**] been called names or insulted? |  |  |  |  |  |
| [**DiscrimMonth9**] been threatened or harassed? |  |  |  |  |  |

***If DiscrimMonth1-9 all = 1 Never, skip to VictimizIntro***

**DiscrimReasonMonth** “In the past 30 days, do you think your experience(s) of being treated with less courtesy than others was related to your…. (Check all that apply)”

1. Age
2. Sex
3. Race
4. Ethnicity
5. Nationality
6. Religion
7. Income Level/Social Class
8. HIV Status
9. Weight
10. Sexual Orientation
11. Gender Identity
12. Gender Expression (how masculine or feminine you appear, i.e., how you walk, talk, or dress)
13. Physical Disability
14. Another Reason (Please Specify: ____________)

**18. ANTI-GENDER VIOLENCE AND VICTIMIZATION**

**Victimiz** “Below is a list of incidents you may have experienced because someone presumed you to be transgender. Please indicate how often you have experienced each incident during the past year.”

|  | In the past year, |
| --- | --- |
|  | How many times have you… (Never=0) |
| [**Victimiz1**] had verbal insults directed at you? |  |
| [**Victimiz2**] been threatened with physical violence? |  |
| [**Victimiz3**] had your personal property damaged or destroyed? |  |
| [**Victimiz4**] had objects thrown at you? |  |
| [**Victimiz5**] been chased or followed? |  |
| [**Victimiz6**] been spit on? |  |
| [**Victimiz7**] been punched, hit, kicked or beaten? |  |
| [**Victimiz8**] been assaulted or wounded with a weapon? |  |
| [**Victimiz9**] been sexually harassed (without assault)? |  |
| [**Victimiz10**] been beaten or assaulted by police? |  |

***If response = 0 for Victimiz1,2,3,4,5,6,7,8,9 AND 10, skip to DiscrimTransIntro.***

**VictimizMed** “Did you get medical care after any of these incidents?”

1 Yes

2 No

**VictimizPolice** “Did you report any of these incidents to the police?”

1 Yes

2 No

**DiscrimTrans** “Below is a list of incidents you may have experienced because someone presumed you to be transgender. Please indicate how often you have experienced each incident during the past year.”

|  | In the past year, |
| --- | --- |
|  | How many times have you… (Never=0) |
| [**DiscrimTrans1**] been denied employment or fired from a job? |  |
| [**DiscrimTrans2**] been denied a promotion or salary increase? |  |
| [**DiscrimTrans3**] received an unfair work evaluation? |  |
| [**DiscrimTrans4**] been evicted or denied housing? |  |
| [**DiscrimTrans5**] been refused services in a bar, restaurant, club, or similar establishment? |  |
| [**DiscrimTrans6**] been refused services in a hotel, motel or similar establishment? |  |
| [**DiscrimTrans7**] been refused other services or accommodations? |  |
| [**DiscrimTrans8**] been denied health insurance? |  |

**19. Physical and Emotional Symptoms Attributed to Mistreatment**

**PhysicalMistreat** “Within the past 30 days, have you experienced any physical symptoms, for example, a headache, an upset stomach, tensing of your muscles, or a pounding heart as a result of how you were treated because someone presumed you to be transgender?”

1 Yes

2 No

3 Don’t Know/Not Sure

4 Prefer Not to Answer

**MentalMistreat** “Within the past 30 days, have you felt emotionally upset, for example angry, sad, or frustrated, as a result of how you were treated because someone presumed you to be transgender?”

1Yes

2 No

3 Don’t Know/Not Sure

4 Prefer Not to Answer

**20. Life Stress (Life Skills)**

**LifeStressIntro** “Stress occurs in many different ways in people’s lives. How often would you say you had problems with…”

1 Never

2 Rarely

3 Sometimes

4 Often

|  |  | In the past year… | In the past 30 days… |
| --- | --- | --- | --- |
| **LifeStress1** | Using transportation |  |  |
| **LifeStress2** | Getting food |  |  |
| **LifeStress3** | Getting adequate clothing |  |  |
| **LifeStress4** | Getting medical care |  |  |
| **LifeStress5** | Finding a safe place to hang out or sleep (housing) |  |  |
| **LifeStress6** | Finding a place to be alone with a sexual partner |  |  |
| **LifeStress7** | Having stuff stolen |  |  |
| **LifeStress8** | Being bothered by the police |  |  |
| **LifeStress9** | Getting a job |  |  |
| **LifeStress10** | Getting what you want or need from a social service agency (such as help accessing public assistance) |  |  |

**21. Stress-Related Growth Scale (Affective Growth Subscale)**

**StressGrowth** “Please indicate how much you AGREE or DISAGREE with the following statements about whether or not your life has changed because of coming to terms with yourself as a transgender or gender nonconforming person.”

1 Strongly Agree

2 Agree

3 Disagree

4 Strongly Disagree

| **StressGrowth1** | I learned to look at things in a more positive way |  |
| --- | --- | --- |
| **StressGrowth2** | I learned better ways to express my feelings |  |
| **StressGrowth3** | I learned not to let hassles bother me the way they used to |  |
| **StressGrowth4** | I learned not to freak out when a bad thing happens |  |
| **StressGrowth5** | I learned to get less angry about things |  |
| **StressGrowth6** | I learned to be a more optimistic person |  |
| **StressGrowth7** | I learned to approach life more calmly |  |

**22. Medical Care experiences**

**MedicalCareIntro** “The next set of questions will ask about your experiences accessing health care.”

**MedExperienceIntro** “In the past 6 months, have you ever had any of the following experiences? (if you NEVER needed medical care in the past 6 months, please check “Not Applicable”)”

1 Yes

2 No

3 Not Applicable

| **MedExperience1** | I postponed or did not try to get medical care when I was sick or injured. |  |
| --- | --- | --- |
| **MedExperience2** | I postponed or did not try to get check-ups or other preventive medical care. |  |
| **MedExperience3** | I postponed or did not try to get medical care when I needed it, and this resulted in a medical emergency where I had to go to the emergency room (ER) or urgent care clinic to get immediate help. |  |
| **MedExperience4** | I had to teach my doctor or other provider about transgender people in order to get appropriate care. |  |
| **MedExperience5** | I was not able to access medical gender affirmation services (i.e., hormones, surgeries for transition). |  |
| **MedExperience6** | A doctor or other provider refused to treat me. |  |

**23. Gender Affirmation and Gender Minority Stress across the HIV Continuum OF CARE**

**GenderAffirmNeedIntro** “The next section contains statements about things that can be done in health care settings to support transgender and gender nonconforming people. In this section, please choose an answer to let us know how important these things have been to you in the past 12 months, whether or not they have actually happened to you."

1 Strongly Disagree

2 Disagree

3 Agree

4 Strongly Agree

| **GenderAffirmNeed1** It is important to me that my preferred name and gender pronouns are always used at the places where I receive health care, including in the waiting room. |  |
| --- | --- |
| **GenderAffirmNeed2** It is important to me that my health care provider apologizes if they make a mistake related to my name or gender pronouns. |  |
| **GenderAffirmNeed3** It is important to me that intake forms at the places where I receive health care ask about my preferred name, my preferred gender pronouns, and my gender identity. |  |
| **GenderAffirmNeed4** It is important to me that my health care provider asks me what words I use for my body parts and describes my body using those words. |  |
| **GenderAffirmNeed5** It is important to me that there is a gender-neutral restroom available at the places where I receive health care. |  |
| **GenderAffirmNeed6** It is important to me that my health care provider is knowledgeable about transgender and gender non-conforming people’s health issues. |  |
| **GenderAffirmNeed7** It is important to me my health care provider is able to provide me resources and referrals that are affirming of my gender identity or gender expression. |  |
| **GenderAffirmNeed8** It is important to me that my health care provider is knowledgeable about working with health insurance in order to meet my health care needs related to my gender identity or gender expression. |  |

**GenderAffirmAccessIntro** “This section contains statements about the same things that can be done in health care settings to support transgender and gender nonconforming people. This time, please choose an answer to let us know how much the following things have actually happened to you when you have received health care in the past 12 months."

1 Strongly Disagree

2 Disagree

3 Agree

4 Strongly Agree

| **GenderAffirmAccess1** My preferred name and gender pronouns are always used at the places where I receive health care, including in the waiting room. |  |
| --- | --- |
| **GenderAffirmAccess2** My health care provider apologizes if they make a mistake related to my name or gender pronouns. |  |
| **GenderAffirmAccess3** Intake forms at the places where I receive health care ask about my preferred name, my preferred gender pronouns, and my gender identity. |  |
| **GenderAffirmAccess4** My health care provider asks me what words I use for my body parts and describes my body using those words. |  |
| **GenderAffirmAccess5** There is a gender-neutral restroom available at the places where I receive health care. |  |
| **GenderAffirmAccess6** My health care provider is knowledgeable about transgender and gender non-conforming people’s health issues. |  |
| **GenderAffirmAccess7** My health care provider is able to provide me resources and referrals that are affirming of my gender identity or gender expression. |  |
| **GenderAffirmAccess8** My health care provider is knowledgeable about working with health insurance in order to meet my health care needs related to my gender identity or gender expression. |  |

**StigmaAnticipated** “The next section contains statements about things you may fear happening when receiving health care. Please choose an answer to let us know whether you have had a fear of the following things happening in the past 12 months."

1 Strongly Disagree

2 Disagree

3 Agree

4 Strongly Agree

| **StigmaAnticipated1** I fear I will hear negative comments about my gender identity or gender expression from others while sitting in the waiting room of the places where I receive health care. |  |
| --- | --- |
| **StigmaAnticipated2** I fear my health care provider will ask me inappropriate questions related to my gender identity or gender expression during a health care visit. |  |
| **StigmaAnticipated3** I fear I will be refused health care because of my gender identity or gender expression. |  |
| **StigmaAnticipated4** I fear I will be offered limited health care services because of my gender identity or gender expression. |  |
| **StigmaAnticipated5** I fear I will hear health care providers or staff gossiping about my gender identity or gender expression during a health care visit. |  |
| **StigmaAnticipated6** I fear a health care provider will refuse to treat me because of my gender identity or gender expression. |  |
| **StigmaAnticipated7** I fear I will be called by the wrong name in the waiting room of the places where I receive health care. |  |
| **StigmaAnticipated8** I fear I will be called by the wrong pronouns in the waiting room of the places where I receive health care. |  |
| **StigmaAnticipated9** I fear I will have to change health care providers because of a negative experience related to my gender identity or gender expression. |  |
| **StigmaAnticipated10** I fear my health care provider will view my gender identity or gender expression as a mental illness. |  |
| **StigmaAnticipated11** I fear my health care provider will make assumptions about my sexual orientation because of my gender identity or gender expression. |  |
| **StigmaAnticipated12** I fear my health care provider will make assumptions about my desire to medically transition (that is, access hormones and/or gender affirmation surgery) due to my gender identity or gender expression. |  |

**StigmaAvoid** “The next section contains statements about these same things you may fear happening when accessing or trying to access health care, but this time we’d like to know whether fear of this happening caused you to avoid needed medical care. Please choose an answer to let us know whether this fear caused you to avoid needed medical care in the past 12 months."

1 Strongly Disagree

2 Disagree

3 Agree

4 Strongly Agree

| **StigmaAvoid1** I have avoided needed care because I fear that I will hear negative comments about my gender identity or gender expression from others while sitting in the waiting room of the places where I receive health care. |  |
| --- | --- |
| **StigmaAvoid2** I have avoided needed care because I fear my health care provider will ask me inappropriate questions related to my gender identity or gender expression during a health care visit. |  |
| **StigmaAvoid3** I have avoided needed care because I fear I will be refused health care because of my gender identity or gender expression. |  |
| **StigmaAvoid4** I have avoided needed care because I fear I will be offered limited health care services because of my gender identity or gender expression. |  |
| **StigmaAvoid5** I have avoided needed care because I fear I will hear health care providers or staff gossiping about my gender identity or gender expression during a health care visit. |  |
| **StigmaAvoid6** I have avoided needed care because I fear a health care provider will refuse to treat me because of my gender identity or gender expression. |  |
| **StigmaAvoid7** I have avoided needed care because I fear I will be called by the wrong name in the waiting room of the places where I receive health care. |  |
| **StigmaAvoid8** I have avoided needed care because I fear I will be called the by the wrong pronouns in the waiting room of the places where I receive health care. |  |
| **StigmaAvoid9** I have avoided needed care because I fear I will have to change health care providers because of a negative experience related to my gender identity or gender expression. |  |
| **StigmaAvoid10** I have avoided needed care because I fear my health care provider will view my gender identity or gender expression as a mental illness. |  |
| **StigmaAvoid11** I have avoided needed care because I fear my health care provider will make assumptions about my sexual orientation because of my gender identity or gender expression. |  |
| **StigmaAvoid12** I have avoided needed care because I fear my health care provider will make assumptions about my desire to medically transition (that is, access hormones and/or gender affirmation surgery) due to my gender identity or gender expression. |  |

**StigmaEnactedIntro** “The next section contains the same statements about things you may fear happening when accessing or trying to access health care, but now we would like to know whether these things have actually happened. Please choose an answer to let us know whether the following things have actually happened to you in the past 12 months."

1 Strongly Disagree

2 Disagree

3 Agree

4 Strongly Agree

| **StigmaEnacted1**I have heard negative comments about my gender identity or gender expression from others while sitting in the waiting room of the places where I receive health care. |  |
| --- | --- |
| **StigmaEnacted2** My health care provider has asked me inappropriate questions related to my gender identity or gender expression during a health care visit. |  |
| **StigmaEnacted3** I have been refused health care because of my gender identity or gender expression. |  |
| **StigmaEnacted4** I have been offered limited health care services because of my gender identity or gender expression. |  |
| **StigmaEnacted5** I have heard health care providers or staff gossiping about my gender identity or gender expression during a health care visit. |  |
| **StigmaEnacted6** A health care provider refused to treat me because of my gender identity or gender expression. |  |
| **StigmaEnacted7** I have been called by the wrong name in the waiting room of the places where I receive health care. |  |
| **StigmaEnacted8** I have been called the by the wrong pronouns in the waiting room of the places where I receive health care. |  |
| **StigmaEnacted9** I have had to change health care providers because of a negative experience related to my gender identity or gender expression. |  |
| **StigmaEnacted10** My health care provider has viewed my gender identity or gender expression as a mental illness. |  |
| **StigmaEnacted11** My health care provider has made assumptions about my sexual orientation because of my gender identity or gender expression. |  |
| **StigmaEnacted12** My health care provider has made assumptions about my desire to medically transition (that is, access hormones and/or gender affirmation surgery) due to my gender identity or gender expression. |  |

**GenderAffirmSettingIntro** “The next questions ask about how supported you have felt in your gender identity or gender expression at the place(s) where you have accessed HIV-related services in the past 12 months. Please rate how supported you felt on a scale from ‘not at all’ supported to ‘very’ supported.”

***If HIVtestever = 0, skip GASetting2-5. If HIVresult = 2,3,11 skip GASetting3-5. (Only ask all 5 settings if participant is living with HIV.)***

| How supported have you felt in your gender identity or gender expression at the place(s) where… |  |  |  |  |
| --- | --- | --- | --- | --- |
| **[GASetting1]**…you have received HIV prevention services? | Not at all | Slightly | Moderately | Very |
| **[GASetting2]**… you have been tested for HIV? | Not at all | Slightly | Moderately | Very |
| **[GASetting3]**… you have received HIV care? | Not at all | Slightly | Moderately | Very |
| **[GASetting4]**… you have gotten your HIV medications? | Not at all | Slightly | Moderately | Very |
| **[GASetting5]**… you have received other support services, such as counseling, case management /care coordination, or HIV education. | Not at all | Slightly | Moderately | Very |

**StigmaSettingIntro** “The next questions ask you about being treated negatively because of your gender identity or gender expression at the place(s) where you have accessed HIV-related services in the past 12 months. The questions ask you to rate how often you have been treated negatively on a scale from ‘never’ treated negatively to ‘very often’ treated negatively.”

***If HIVtestever = 0, skip StigmaSetting2-5. If HIVresult = 2,3,11 skip StigmaSetting3-5. (Only ask all 5 settings if participant is living with HIV.)***

| How often have you been treated negatively because of your gender identity or gender expression at the place(s) where… |  |  |  |  |  |
| --- | --- | --- | --- | --- | --- |
| **[StigmaSetting1]**… you have received HIV prevention services? | Never | Hardly ever | Not Too Often | Fairly Often | Very Often |
| **[StigmaSetting2]**… you have been tested for HIV? | Never | Hardly ever | Not Too Often | Fairly Often | Very Often |
| **[StigmaSetting3]**… you have received HIV care? | Never | Hardly ever | Not Too Often | Fairly Often | Very Often |
| **[StigmaSetting4]**… you get your HIV medications? | Never | Hardly ever | Not Too Often | Fairly Often | Very Often |
| **[StigmaSetting5]**… you receive other support services, such as counseling, case management /care coordination, or HIV education. | Never | Hardly ever | Not Too Often | Fairly Often | Very Often |

**24. PRIOR INVOLVEMENT IN PROGRAMS OR RESEARCH STUDIES**

**ProgramIntro** “The next set of questions will ask about your experiences participating in other research studies, programs, or support groups.”

**ProgramInvolvement** “Other than this study, ATN 130, have you ever participated in any programs, interventions, research studies, support groups or individual counseling sessions about HIV, sexual risk, relationships, gender identity, gender expression, or sexuality?”

1 Yes

2 No ***(Skip to EndScript)***

**ProgramNumber** “How many programs, interventions, research studies, support groups or individual counseling sessions about HIV, sexual risk, relationships, gender identity, gender expression, or sexuality have you participated in?”

**|_____|_____|**

**ProgramDetails1** “What did the session(s) or meeting(s) you attended cover? (Check all that apply)”

1. Sexual risk reduction
2. Reducing substance use
3. Disclosure of HIV status
4. Treatment engagement
5. Medication adherence
6. Mental health/well-being
7. Empowerment
8. Oppression/Stigma/Discrimination
9. Relationships
10. Family
11. Gender identity/gender expression
12. Unknown
13. Other, specify: _____________________

**ProgramDetails2** “Were any of these programs specifically for transgender and/or gender nonconforming young people?”

1 Yes

2 No

**ProgramDetails3** “Were any of these programs limited to only [%CurrGender%] participants?”

1 Yes

2 No

**EndScript** “Thank you for your participation. Please let the interviewer know that you are finished.”
